# *PDXK* mutations cause polyneuropathy responsive to pyridoxal 5′‐phosphate supplementation

**DOI:** 10.1002/ana.25524

**Published:** 2019-07-01

**Authors:** Viorica Chelban, Matthew P. Wilson, Jodi Warman Chardon, Jana Vandrovcova, M. Natalia Zanetti, Eleni Zamba‐Papanicolaou, Stephanie Efthymiou, Simon Pope, Maria R. Conte, Giancarlo Abis, Yo‐Tsen Liu, Eloise Tribollet, Nourelhoda A. Haridy, Juan A. Botía, Mina Ryten, Paschalis Nicolaou, Anna Minaidou, Kyproula Christodoulou, Kristin D. Kernohan, Alison Eaton, Matthew Osmond, Yoko Ito, Pierre Bourque, James E. C. Jepson, Oscar Bello, Fion Bremner, Carla Cordivari, Mary M. Reilly, Martha Foiani, Amanda Heslegrave, Henrik Zetterberg, Simon J. R. Heales, Nicholas W. Wood, James E. Rothman, Kym M. Boycott, Philippa B. Mills, Peter T. Clayton, Henry Houlden, for the Care4Rare Canada Consortium and the SYNaPS Study Group, Yamna Kriouile, Mohamed El Khorassani, Mhammed Aguennouz, Stanislav Groppa, Blagovesta Marinova Karashova, Lionel Van Maldergem, Wolfgang Nachbauer, Sylvia Boesch, Larissa Arning, Dagmar Timmann, Bru Cormand, Belen Pérez‐Dueñas, Gabriella Di Rosa, Jatinder S. Goraya, Tipu Sultan, Jun Mine, Daniela Avdjieva, Hadil Kathom, Radka Tincheva, Selina Banu, Mercedes Pineda‐Marfa, Pierangelo Veggiotti, Michel D. Ferrari, Arn M J M van den Maagdenberg, Alberto Verrotti, Giangluigi Marseglia, Salvatore Savasta, Mayte García‐Silva, Alfons Macaya Ruiz, Barbara Garavaglia, Eugenia Borgione, Simona Portaro, Benigno Monteagudo Sanchez, Richard Boles, Savvas Papacostas, Michail Vikelis, James Rothman, Paola Giunti, Henry Houlden, Viorica Chelban, Vincenzo Salpietro, Emer Oconnor, Stephanie Efthymiou, Dimitri Kullmann, Rauan Kaiyrzhanov, Roisin Sullivan, Alaa Matooq Khan, Wai Yan Yau, Isabel Hostettler, Eleni Zamba Papanicolaou, Efthymios Dardiotis, Shazia Maqbool, Shahnaz Ibrahim, Salman Kirmani, Nuzhat Noureen Rana, Osama Atawneh, Shen‐Yang Lim, Farooq Shaikh, George Koutsis, Marianthi Breza, Salvatore Mangano, Carmela Scuderi, Eugenia Borgione, Giovanna Morello, Tanya Stojkovic, Erin Torti, Massimi Zollo, Gali Heimer, Yves A. Dauvilliers, Pasquale Striano, Issam Al‐Khawaja, Fuad Al‐Mutairi, Fowzan S Alkuraya, Hamed Sherifa, Mie Rizig, Njideka U. Okubadejo, Oluwadamilola O. Ojo, Olajumoke O. Oshinaike, Kolawole Wahab, Abiodun H. Bello, Sanni Abubakar, Yahaya Obiabo, Ernest Nwazor, Oluchi Ekenze, Uduak Williams, Alagoma Iyagba, Lolade Taiwo, Morenikeji Komolafe, Olapeju Oguntunde, Sofya Pchelina, Konstantin Senkevich, Nourelhoda Haridy, Chingiz Shashkin, Nazira Zharkynbekova, Kairgali Koneyev, Ganieva Manizha, Maksud Isrofilov, Ulviyya Guliyeva, Kamran Salayev, Samson Khachatryan, Salvatore Rossi, Gabriella Silvestri, Thomas Bourinaris, Georgia Xiromerisiou, Liana Fidani, Cleanthe Spanaki, Arianna Tucci

**Affiliations:** ^1^ Department of Neuromuscular Diseases University College London Queen Square Institute of Neurology London United Kingdom; ^2^ Department of Neurology and Neurosurgery Institute of Emergency Medicine Chisinau Moldova; ^3^ Genetics and Genomic Medicine University College London Great Ormond Street Institute of Child Health London United Kingdom; ^4^ Department of Medicine (Neurology) University of Ottawa Ottawa Ontario Canada; ^5^ Ottawa Hospital Research Institute Ottawa Ontario Canada; ^6^ Children's Hospital of Eastern Ontario Research Institute, University of Ottawa Ottawa Ontario Canada; ^7^ Department of Clinical and Experimental Epilepsy University College London Queen Square Institute of Neurology London United Kingdom; ^8^ Cyprus Institute of Neurology and Genetics Nicosia Cyprus; ^9^ Cyprus School of Molecular Medicine Nicosia Cyprus; ^10^ Neurometabolic Unit, National Hospital for Neurology and Neurosurgery London United Kingdom; ^11^ Randall Centre of Cell and Molecular Biophysics, School of Basic and Medical Biosciences King's College London London United Kingdom; ^12^ Department of Neurology Neurological Institute, Taipei Veterans General Hospital Taipei Taiwan; ^13^ National Yang‐Ming University School of Medicine Taipei Taiwan; ^14^ Institute of Brain Science, National Yang‐Ming University Taipei Taiwan; ^15^ Department of Neurology and Psychiatry Assiut University Hospital, Faculty of Medicine Asyut Egypt; ^16^ Reta Lila Weston Research Laboratories University College London Queen Square Institute of Neurology London United Kingdom; ^17^ Department of Information and Communications Engineering University of Murcia Murcia Spain; ^18^ Department of Medical & Molecular Genetics King's College London, Guy's Hospital London United Kingdom; ^19^ Newborn Screening Ontario, Children's Hospital of Eastern Ontario Ottawa Ontario Canada; ^20^ Neuro‐ophthalmology Department National Hospital for Neurology and Neurosurgery London United Kingdom; ^21^ Clinical Neurophysiology Department National Hospital for Neurology and Neurosurgery London United Kingdom; ^22^ Department of Neurodegenerative Disease University College London Queen Square Institute of Neurology London United Kingdom; ^23^ UK Dementia Research Institute at University College London London United Kingdom; ^24^ Clinical Neurochemistry Laboratory Sahlgrenska University Hospital Mölndal Sweden; ^25^ Department of Psychiatry and Neurochemistry Institute of Neuroscience and Physiology, Sahlgrenska Academy at University of Gothenburg Mölndal Sweden; ^26^ Neurogenetics Laboratory National Hospital for Neurology and Neurosurgery London United Kingdom; ^27^ Department of Cell Biology Yale School of Medicine New Haven CT

## Abstract

**Objective:**

To identify disease‐causing variants in autosomal recessive axonal polyneuropathy with optic atrophy and provide targeted replacement therapy.

**Methods:**

We performed genome‐wide sequencing, homozygosity mapping, and segregation analysis for novel disease‐causing gene discovery. We used circular dichroism to show secondary structure changes and isothermal titration calorimetry to investigate the impact of variants on adenosine triphosphate (ATP) binding. Pathogenicity was further supported by enzymatic assays and mass spectroscopy on recombinant protein, patient‐derived fibroblasts, plasma, and erythrocytes. Response to supplementation was measured with clinical validated rating scales, electrophysiology, and biochemical quantification.

**Results:**

We identified biallelic mutations in *PDXK* in 5 individuals from 2 unrelated families with primary axonal polyneuropathy and optic atrophy. The natural history of this disorder suggests that untreated, affected individuals become wheelchair‐bound and blind. We identified conformational rearrangement in the mutant enzyme around the ATP‐binding pocket. Low PDXK ATP binding resulted in decreased erythrocyte PDXK activity and low pyridoxal 5′‐phosphate (PLP) concentrations. We rescued the clinical and biochemical profile with PLP supplementation in 1 family, improvement in power, pain, and fatigue contributing to patients regaining their ability to walk independently during the first year of PLP normalization.

**Interpretation:**

We show that mutations in *PDXK* cause autosomal recessive axonal peripheral polyneuropathy leading to disease via reduced PDXK enzymatic activity and low PLP. We show that the biochemical profile can be rescued with PLP supplementation associated with clinical improvement. As B_6_ is a cofactor in diverse essential biological pathways, our findings may have direct implications for neuropathies of unknown etiology characterized by reduced PLP levels. ANN NEUROL 2019;86:225–240

Peripheral neuropathies are among the most common neurological disorders worldwide, affecting approximately 20 million people in Europe and the USA alone, with few disease‐modifying treatments established for these conditions.^1^, ^2^, ^3^ Inherited forms of peripheral neuropathies affecting both the motor and sensory nerves, known as Charcot–Marie–Tooth disease (CMT), are the most common genetic neuromuscular disorders, affecting approximately 1 in 2,500 people.^4^, ^5^, ^6^, ^7^, ^8^ However, only 25% of all autosomal recessive (AR) CMT cases have causal variants identified,^9^ and treatment is only supportive.^1^, ^2^, ^3^ Most known genes associated with AR CMT cause disease either by affecting the cytoskeleton or axonal trafficking.^2^

## Subjects and Methods

### *Study Participants*

Patients with polyneuropathy and optic atrophy were identified by neurogeneticists at the National Hospital for Neurology and Neurosurgery London and Children's Hospital of Eastern Ontario. The affected cases were recruited along with unaffected family members under institutional review board/ethics‐approved research protocols (University College London Hospitals: 04/N034; Children's Hospital of Eastern Ontario: CTO/1577) with informed consent. All cases had extensive genetic, metabolic, and mitochondrial investigations carried out that excluded acquired and other inherited causes of polyneuropathy and optic atrophy.

### *Phenotype and Clinical Measures*

All cases had comprehensive phenotyping performed by neurogenetics specialists including clinical assessment, electrophysiology, neuroimaging, and videography. Motor function was quantified using the Medical Research Council (MRC) muscle scale, a standardized scale for the assessment of the peripheral nervous system (PNS) that grades muscle power from 0 to 5 in 6 validated movements and provides a total score of a possible 60 points. The disease severity and progression were assessed using the validated Neurological Impairment Scale (NIS)^10^ and the Charcot‐Marie‐Tooth Neuropathy Score version 2 (CMTNS2). CMTNS2 is a reliable and valid composite of symptoms (3 items), signs (4 items), and neurophysiology (2 items). It is designed to measure length‐dependent motor and sensory impairment in genetic neuropathies.^11^, ^12^ The NIS forms part of the standard minimum dataset for the Functional Independence Measure and UK Functional Assessment Measure. It records the severity of functional impairment (rated 0–3) across 13 domains mapped onto the International Classification of Functioning. The score range is 0 to 50. The affected individuals from Family 1 had MRC scales, CMTNS2, and NIS assessment before and after replacement therapy.

### *Genome‐wide Sequencing and Haplotype Analysis*

DNA was extracted from peripheral blood. Whole genome sequencing (WGS) was performed by deCODE genetics (Reykjavík, Iceland), using DNA from the 2 affected siblings from Family 1. A HiSeq4000 instrument (Illumina, San Diego, CA) was used to generate 100bp paired‐end reads. Alignment was performed using BWA (http://bio-bwa.sourceforge.net/)^13^ with GRCH37 as a reference. Variants were called using the GATK^14^, ^15^, ^16^, ^17^ Unified Genotyper‐based pipeline^14^, ^15^, ^16^ workflow. All variants were annotated using ANNOVAR^18^ and filtered using custom R scripts. Shared regions of homozygosity were identified using HomozygosityMapper^19^ and Bcftools/RoH.^20^ Only novel or very rare variants with a minor allele frequency of <0.01 in the 1000 Genomes Project,^21^ NHLBI GO Exome Sequencing,^22^ and Exome Aggregation Consortium database^23^ were included. Coding/splicing homozygous variants that were within the autozygome of the affected cases were prioritized.

Whole exome sequencing (WES) was performed in Family 2 using DNA from both affected siblings and their unaffected mother as part of the Care4Rare Canada Consortium. Exonic DNA was selected using the SureSelect Clinical Research Exome V1 kit (Agilent Technologies, Santa Clara, CA), then sequenced on an Illumina NextSeq 500 with 2 × 150bp chemistry. Read alignment, variant calling, and annotation were done as outlined for previous FORGE and Care4Rare Canada projects.^24^ Average coverage for the exomes was 182×, 190×, and 244×, and >97% of consensus coding sequence exons in all exomes were covered at >10×.

### *Sanger Sequencing*

The variants identified by WGS and WES were confirmed by Sanger sequencing. The region was amplified using the following primers: 5′‐AGGAGGATCAGGGATGGGAG‐3′ and 5′‐CTCTCATATCCTGCTCCCCA‐3′ in Family 1 and 5′‐CTTGCCGCTCTATGTTTGGAC‐3′ and 5′‐GAGAGTGACAGGCGCAACTC‐3′ in Family 2. The promoter region of *PDXK* was amplified using the following primers: 5′‐GCGGTTCCCTTGGGTATC‐3′ and 5′‐ACGCCTCCTTCTGACCTC‐3′. Genomic DNA was extracted from dried blood spots (DBSs) from affected individuals from Family 1 and controls using the QIAamp DNA Micro Kit (Qiagen, Valencia, CA) and from blood from Family 2. Sequencing reactions were performed using the BigDye Terminator 1.1 system (Thermo Fisher Scientific, Paisley, UK) followed by sequencing using an ABI DNA Analyser (Thermo Fisher Scientific). Electropherograms were analyzed using the Sequencher software package (Gene Codes Corporation, Ann Arbor, MI).

### *Gene Co‐expression Analysis*

We generated gene co‐expression networks for central nervous system (CNS) and PNS tissue‐specific transcriptomic data generated by the Genotype‐Tissue Expression Consortium (GTEx7 V6 gene expression data are accessible from https://www.gtexportal.org/).^25^ We analyzed each of the tissue sample sets separately by filtering genes on the basis of an Reads Per Kilobase Million (RPKM) >0.1 (observed in >80% of the samples for a given tissue). We then corrected for batch effects, age, gender, and RNA integrity numbers (RIN) using ComBat.^26^ Finally, we used the residuals of these linear regression models to construct gene co‐expression networks for each tissue using WGCNA^27^ and postprocessing with k‐means^28^ to improve gene clustering. Gene modules were functionally annotated with gProfileR4 R package using the Gene Ontology (GO) database without electronic inferred annotations and accounting for multiple testing with gSCS.^29^ The top‐down plot figure was done with Cytoscape 3.5.1.^30^ We initially selected the 50 most connected genes within the black module, for the coexpression network of tibial nerve, based on adjacency values. We included all connections between genes. Then we filtered out the connections with weight <0.05. Finally, we applied a Kamada–Kawai layout algorithm to the remaining genes and edges to dispose them spatially at the canvas so their relative positions reflected similarity in connections.

### *Plasmid Constructs*

The human PDXK wild‐type (WT) and PDXK mutant p.Ala228Thr cDNA were purchased from Invitrogen (Carlsbad, CA) and cloned into the plasmid pGEX6p‐1 (GE Healthcare, Piscataway, NJ) using the sites BamHI and NotI (New England Biolabs, Ipswich, MA).

### *Protein Purification*

*Escherichia coli* strain Rosetta2 (DE3; Novagen, Darmstadt, Germany) was transformed with the plasmids pGEX6p‐1‐PDXK and pGEX6p‐1‐PDXK p.Ala228Thr and grown at 37°C to an OD_600nm_ of 0.8 and then induced with 0.5mM isopropyl β‐D‐1‐thiogalactopyranoside for 4 hours. Cells were lysed, using a cell disruptor (Avestin, Ottawa, Ontario, Canada), in lysis buffer containing 25mM hydroxyethylpiperazine ethane sulfonic acid (HEPES), pH 7.4, 400mM KCl, 4% Triton X‐100, 1mM 1,4‐dithiothreitol (Sigma‐Aldrich, Gillingham, UK), and SIGMAFAST Protease Inhibitor Cocktail Tablets, EDTA‐Free (Sigma‐Aldrich). The samples were supplemented with 5% polyethylamine (Sigma‐Aldrich) and clarified using a 45Ti rotor (Beckman Coulter, Brea, CA) at 100,000 × *g* for 30 minutes prior to incubation overnight at 4°C with glutathione agarose beads (Pierce, Rockford, IL) previously washed in lysis buffer containing 1% Triton X‐100. The glutathione agarose beads containing the immobilized proteins GST‐PDXK and PDXK p.Ala228Thr were subsequently washed in lysis buffer containing 1% Triton X‐100 and incubated with 10mg/ml DNAse and RNAse (Sigma‐Aldrich) for 1 hour at room temperature (RT), prior to washing in lysis buffer containing no Triton X‐100. The GST tag was cleaved using 100U of PreScission Protease (GE Healthcare) for 2 hours at RT. After elution, the proteins were dialyzed in 25mM HEPES, pH 7.4, 100mM KCl, 1mM 1,4‐dithiothreitol overnight at 4°C. Protein concentration was determined using the BCA Protein Assay Kit (Thermo Fisher Scientific).

### *Circular Dichroism*

To investigate conformational differences between WT PDXK and the disease‐linked mutant p.Ala228Thr, circular dichroism (CD) analyses were performed. The extinction coefficients of both proteins (ε_280_ = 31,400M^−1^cm^−1^) were calculated using the ProtParam tool within the ExPASy Portal.^31^ Proteins were prepared as described above and dialyzed overnight at 4°C in 30mM tris, pH 7, 100mM KCl, 3mM MgCl_2_, 0.5mM tris(2‐carboxyethyl)phosphine (TCEP). Samples were concentrated to 0.2mg/ml using Amicon Ultra 0.5ml centrifugal filters (Merck, Darmstadt, Germany).

Ultraviolet (UV) and CD spectra were acquired on the Applied Photophysics (Leatherhead, UK) Chirascan Plus spectrometer across the 400 to 190nm wavelength region. Ten‐millimeter (400–230nm) and 0.5mm (260–190nm) strain‐free rectangular cells were employed. The instrument was flushed continuously with pure evaporated nitrogen throughout the experiment. The following parameters were employed: 2nm spectral bandwidth, 1nm step size, and 1‐second instrument measurement time‐per‐point. All spectra were acquired at 25°C, and buffer baseline corrected. Light scattering correction was applied on the UV spectra of both proteins using the Chirascan Pro‐Data Software (Applied Photophysics). Where possible, spectra were smoothed with a window factor of 4 using the Savitzky–Golay method^32^ for better presentation.

The far‐UV CD spectra of the 2 proteins were corrected for concentration and pathlength and expressed in terms of Δε (M^−1^cm^−1^) per amino acid residue. Protein secondary structure content was assessed using the Principle Component Regression method based on 16 known protein structures^31^ embedded in the PLSPlus/IQ routine in GRAMS32 AI software (Galactic Software, Salem, MA).

### *Isothermal Titration Calorimetry*

The affinity of a nonhydrolyzable analogue of adenosine triphosphate (ATP), called adenosine 5′‐(3‐thiotriphosphate) tetralithium salt (ATPγS), for both PDXK WT and p.Ala228Thr mutant, was examined using a MicroCal Isothermal Titration Calorimeter ITC200 instrument (Malvern Panalytical, Malvern, UK). Both proteins were prepared as described above in 30mM tris, pH 7, 100mM KCl, 3mM MgCl_2_, 0.5mM TCEP (isothermal titration calorimetry [ITC] buffer). An initial 0.2M stock of ATPγS was prepared in water and diluted with ITC buffer to a final concentration of 250μM. The concentration was adjusted using the UV absorbance at 259nm, and the extinction coefficient of 15.4mM^−1^cm^−1^ suggested by the ligand provider (Jena Bioscience, Jena, Germany).

The experiments were conducted at 25°C following standard procedures as described previously.^33^ Forty microliters of 250μM ATPγS was titrated into a 330μl solution of 25μM WT or p.Ala228Thr protein. The ITC experiment consisted of twenty 2μl injections with a spacing of 180 seconds. Heat produced by titrant dilution was obtained by a control experiment, titrating into buffer alone, under the same conditions.

The MicroCal‐Origin 7.0 software package was used to fit the integrated heat data obtained for the titrations corrected for heats of dilution, using a nonlinear least‐squares minimization algorithm based on an independent binding sites model. ΔH (reaction enthalpy change in kcal/mol), Ka (1/K_D_; equilibrium association constant per molar), and n (molar ratio of the proteins in the complex) were the fitting parameters. The reaction entropy was calculated using the relationships ΔG = ΔH − TΔS = −RTln(1/K_D_).

### *Western Blotting*

Fibroblast samples from affected, carriers, and healthy people were obtained with consent. After reaching 90% confluence, the cultured cells were harvested and lysed in ice‐cold lysis radioimmunoprecipitation assay buffer containing 50mM tris‐HCl pH 8.0, 150mM NaCl, 0.5% DOC, 0.1% Triton X‐100, 0.1% sodium dodecyl sulphate (SDS), and SIGMAFAST Protease Inhibitor Cocktail for 30 minutes. The lysate was then centrifuged at 16,000 × *g* at 4°C, and the supernatant transferred to a new tube. The protein concentration was measured using the BCA Protein Assay Kit (Thermo Fisher Scientific) according to manufacturer's protocol. Equal amounts of protein (30μg) from affected and healthy individuals were separated on a 10% SDS–polyacrylamide gel electrophoresis gel (NuPAGE, Invitrogen) and transferred onto an Immobilon membrane (Millipore, Billerica, MA). After blocking the membrane with 2% fat‐free milk in phosphate‐buffered saline–Tween for 1 hour at RT, the membrane was incubated with anti‐PDXK antibody (Abcam, Cambridge, UK; 38208, 1:500) for 1 hour at RT. The membranes were subsequently washed and incubated with horseradish peroxidase–conjugated goat antirabbit IgG (Bio‐Rad Laboratories, Hercules, CA; 17210, 1:5,000) for 1 hour at RT. Blots were developed using ECL Prime (GE Healthcare), visualized via a ChemiDoc Touch Imaging System (Bio‐Rad Laboratories), and analyzed using Image Lab 5.2 software (Bio‐Rad Laboratories). For the quantification, the signal intensity of each band corresponding to the PDXK WT and PDXK p.Ala228Thr mutant protein was normalized to the signal intensity of the corresponding reversible Ponceau staining as a reliable loading control.^34^ The amount of PDXK WT and mutant protein was expressed as a percentage of the control sample. The experiment was repeated 3 times.

### *Measurement of Pyridoxal Kinase Activity*

The pyridoxal (PL) kinase activity present in DBSs was determined by adapting the protocol used by Wilson et al,^35^ with the following modifications. Three‐millimeter discs punched from DBSs were incubated for 10 minutes at 37°C with shaking at 300rpm in a reaction buffer containing 20mmol/l potassium phosphate adjusted to pH 6.1, 10μmol/l PL, and 300μmol/l MgATP (all purchased from Sigma‐Aldrich). The same method was adapted for the investigation of activity of the purified recombinant PL kinase. Five microliters of 20ng/μl purified PL kinase protein lysate was placed in an ultraperformance liquid chromatography (UPLC) 96‐well sample plate (Waters, Elstree, UK). Subsequently, 115μl of reaction buffer containing potassium phosphate (pH 6.1), PL, and MgATP was added to create a final concentration of 20mmol/l potassium phosphate, with PL and MgATP concentrations adjusted as appropriate. Investigation of PL kinase kinetics as a function of PL concentration used an MgATP concentration of 300μmol/l. A PL concentration of 50μmol/l was utilized for the determination of PL kinase kinetics as a function of MgATP concentration. After reaction buffer addition, the plate was incubated for 10 minutes at 37°C in an Eppendorf (Hamburg, Germany) Thermomixer C with shaking at 300rpm prior to addition of 120μl of a reaction stop mix identical to that used for the determination of pyridoxamine (PM) 5′‐phosphate (PMP) oxidase activity from DBSs.

DBSs were collected from 4 affected cases and 22 controls (age = 15–92 years). PL kinase activity of the recombinant proteins and that of DBSs were determined by using UPLC–tandem mass spectrometry (MS/MS) to determine the formation of PL 5′‐phosphate (PLP) after incubation of these with the enzyme substrate PL and expressed as μmol PLP l^−1^h^−1^ from recombinant protein and as pmol PLP (3mm DBS)^−1^h^−1^ from DBSs.

Data collection and statistical analysis were performed using Waters MassLynx and GraphPad (San Diego, CA) Prism 6.0 software packages.

### *UPLC‐MS/MS Measurement of the B_6_ Vitamers*

B_6_ vitamer concentrations (pyridoxine, PL, PLP, PM, PMP) and pyridoxic acid were quantified by UPLC‐MS/MS using stable isotope‐labeled internal standards.^35^, ^36^

### *High‐Performance Liquid Chromatograph Measurement of B_6_ Vitamers*

PL phosphate was measured by high‐performance liquid chromatography (HPLC) with fluorescence detection using a Chromsystems Instruments & Chemicals (Gräfelfing, Germany) kit as described previously.^35^ Pyridoxic acid concentration was calculated using an external calibration standard.

### *PLP Measurements before and after PLP Replacement*

Plasma PLP concentrations before and after PLP replacement were measured using 2 independent HPLC and UPLC‐MS/MS methods as described above to validate the results.

The difference between groups was tested with the use of a 1‐way analysis of variance test, followed by Tukey–Kramer test.

### *Measurement of Neurofilament Light Chain Concentration*

Plasma neurofilament light chain (NFL) concentration was measured by digital enzyme‐linked immunosorbent assay using the commercially available NF‐Light kit on a single molecule array (Simoa) platform (HD‐1 Analyzer) according to instructions by the manufacturer (Quanterix, Lexington, MA). All measurements were performed in duplicates, in 1 round of experiments using 1 batch of reagents. Intra‐assay coefficients of variation were <10%.

## Results

**Figure 1:**
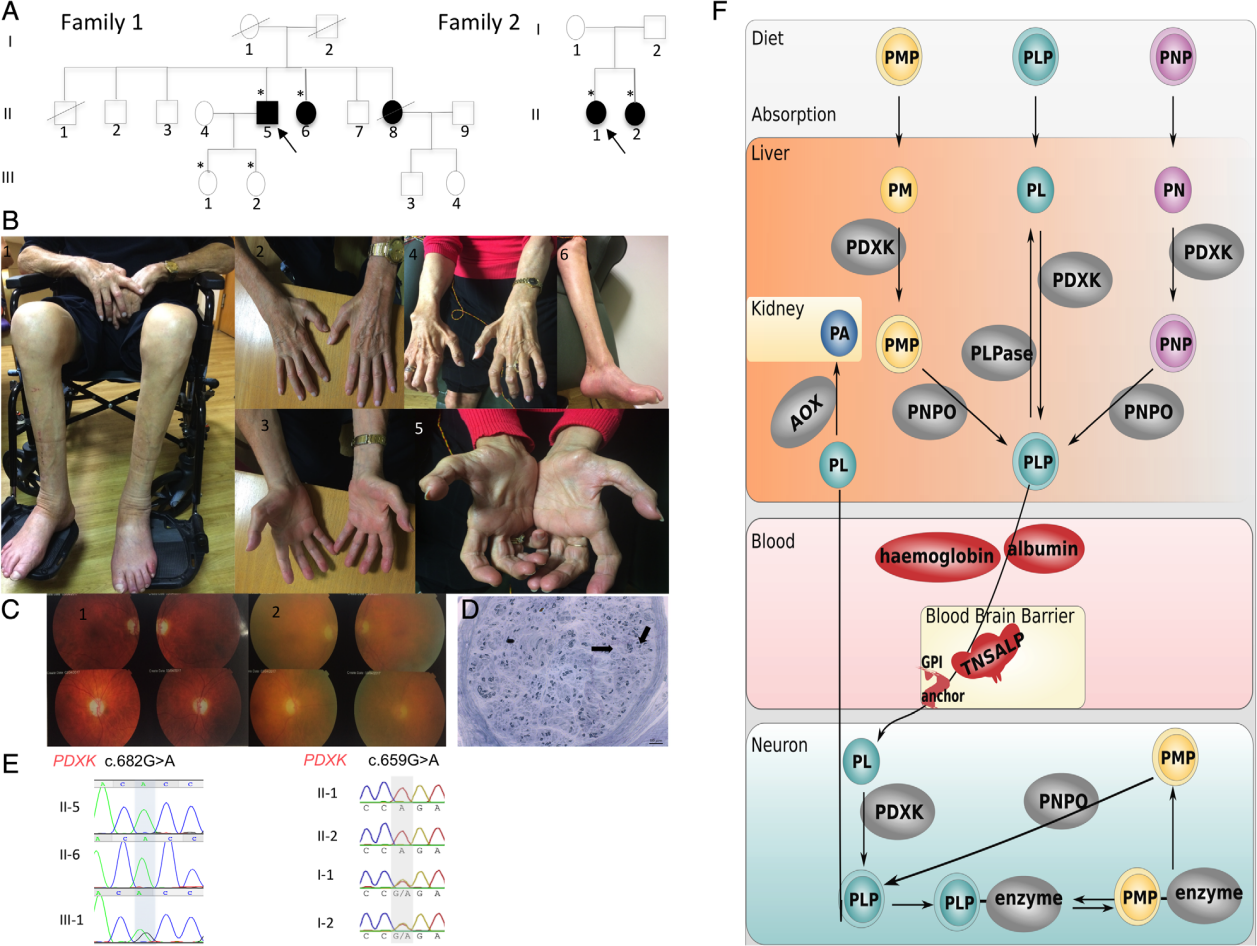
Biallelic *PDXK* mutations are associated with axonal polyneuropathy and optic atrophy. (A) Pedigree of the 2 families with *PDXK* mutations. * = individuals examined. Arrow = proband. (B) Phenotype of the cases with *PDXK* mutations presenting with muscle atrophy of the intrinsic muscles of the hands, with clawing of the hands, thin wrists, pes cavus, and muscle atrophy in the feet and calves (B1‐B3, Case F1‐II‐5; B4‐B6, Case F1‐II‐6). (C) Fundoscopy‐confirmed bilateral optic disc atrophy in both cases (C1, F1‐II‐5; C2, F1‐II‐6). (D) Nerve biopsy from Case F1‐II‐6 showing axonopathy. There is diffuse and severe depletion of both small and large myelinated axons with most of the surviving axons being <5μm in diameter. There were no axonal ovoids, but there were regenerating clusters *(arrows)* consistent with longstanding indolent axonopathy. There was no demyelination process. (E) Sanger sequencing confirming the homozygous c.682G>A mutation in the 2 affected siblings and heterozygous state in an unaffected family member in Family 1, and homozygous c.659G>A in the 2 affected siblings and segregation in Family 2. (F) Vitamin B_6_ metabolic pathway. Phosphorylated B_6_ vitamers (pyridoxamine 5′‐phosphate [PMP]; pyridoxine 5′‐phosphate [PNP]; pyridoxal 5′‐phosphate [PLP]) present in the diet are hydrolyzed to pyridoxal (PL), pyridoxamine (PM), and pyridoxine (PN) by intestinal phosphatases prior to absorption and then converted to their 5′‐phosphate derivatives in the liver by PL kinase (PDXK). PNP and PMP are then converted to PLP by pyridox(am)ine 5′‐phosphate oxidase (PNPO). PLP re‐enters the circulation bound to a lysine residue of albumin. Homeostatic regulation of tissue levels of PLP is achieved by various mechanisms, including feedback inhibition of PNPO and PL kinase by PLP.^62^ Albumin in plasma, hemoglobin in erythrocytes, and glycogen phosphorylase in muscle also play a role, binding to PLP and helping to keep concentrations of this very reactive aldehyde low^63^ so as to avoid any unwanted reactions with biologically important molecules. Subsequent delivery of PLP to the tissues requires hydrolysis of circulating PLP to PL by the ectoenzyme tissue nonspecific alkaline phosphatase (TNSALP). The resulting pyridoxal is able to enter cells prior to being rephosphorylated by PL kinase to produce the active cofactor, PLP, required by B_6_‐dependent apoenzymes.^64^ Within cells, recycling pathways also exist, with PMP being oxidized by PNPO to form PLP.^63^ AOX = aldehyde oxidase; GPI = glycosylphosphatidylinositol; PA = pyridoxic acid.

Here, we describe 5 affected cases from 2 unrelated families (Fig 1A) with early, childhood onset, sensorimotor, length‐dependent, predominantly axonal polyneuropathy and adult onset optic atrophy (Table 1).

**Table 1:** Detailed Phenotype of the Affected Cases Carrying *PDXK* Mutations

Phenotype/Case	F1‐II‐5	F1‐II‐6	F1‐II‐8	F2‐II‐1	F2‐II‐2
Demographics					
Gender	M	F	F	F	F
Age at examination, yr	79	74	Died at 71 years from leukemia	31	29
Age at onset, yr	7	9	7	5	2
Progression					
Symptoms at onset	Lower limb weakness and wasting				
Upper limb weakness	At 12 years	At 12 years	At 17 years	20s	20s
Optic atrophy	At 40 years	At 47 years	At 50 years	31	29
Neurological examination					
Fundoscopy	Pale optic discs bilaterally	Pale optic discs bilaterally	NA	Mild optic disc pallor bilaterally	Mild optic disc pallor bilaterally
Other cranial nerves	Normal				
Skeletal deformities	Pes cavus, hammer toes, clawing of hands			Pes cavus, hammer toes	
Power	Severe weakness of dorsiflexion/plantar flexion, long finger extensors and intrinsic muscles of the hands			Moderate–severe weakness of dorsiflexion/plantar flexion, long finger extensors and mild weakness of intrinsic muscles of the hands	
MRC power score	44	44		56	54
Reflexes	Absent throughout; mute plantar responses		NA	Areflexia; mute plantar responses	Areflexia; mute plantar responses
Sensation	Reduced pain to midcalf and wrists	Reduced pain to ankles and elbows		Reduced pain to ankles	Reduced pain to upper calf and base of fingers
	Reduced vibration sense to iliac crest and wrist	Reduced vibration sense to iliac crest and elbow		Vibration decreased to the knees	Vibration decreased to the knees
Romberg sign	Present	Present		Present	Present
Coordination	Normal	Normal		Normal	Normal
Visual acuities	Unable to count fingers	6/12 in both eyes		NA	NA
Color vision	Unable to distinguish any color	Grossly impaired, 3/17 Ishihara plates		15/17 Ishihara plates	3/17 Ishihara plates
Peripheral vision	Normal	Normal		Normal	Normal
Cognitive function	Normal	Normal		Normal	Normal
Seizures	Absent	Absent		Absent	Absent
Investigation results					
MRI head	Normal	Normal		NA	NA
Optic nerve and chiasm CT	Normal	Normal		NA	NA
VEPs	Severely attenuated bilaterally with anomalous waveform on flash VEPs			NA	NA
Somatosensory evoked potentials	NA	Poorly formed due to severe polyneuropathy		NA	NA
Nerve conduction study	Severe sensorimotor axonal neuropathy	Severe sensorimotor axonal neuropathy		Severe sensorimotor axonal neuropathy	Severe sensorimotor axonal neuropathy
Electromyography	Chronic denervation in a length‐dependent pattern; no myopathic changes			Chronic denervation in a length‐dependent pattern; no myopathic changes	
Renal function	Normal	Normal		Normal	Normal
Biochemical profile					
Liver function	Normal	Normal		Normal	Normal
GI tract	Normal colonoscopy	Normal		Normal	Normal
Plasma amino acids	Normal	NA		NA	NA
Vitamin B_1_, normal range = 67–265nmol/l	NA	176		149	183
Vitamin B_9_, normal range = 3.9–20ng/ml	Normal	NA		Normal	Normal
Vitamin B_12_, normal range = 197–771pg/ml	938	454		293	211

Plasma amino acids tested: methionine, isoleucine, leucine, tyrosine, phenylalanine, ornithine, lysine, histidine, and arginine.

CT = computed tomography; F = female; GI = gastrointestinal; M = male; MRC = Medical Research Council; MRI = magnetic resonance imaging; NA = not available; VEP = visual evoked potential.

### *Disease Characterization*

Family 1 is of Cypriot origin (affected individuals F1‐II‐5, currently 80 years old, F1‐II‐6, currently 75 years old, and F1‐II‐9, died at 71 years old from an unrelated medical condition). Three affected siblings presented with difficulties running, distal wasting, and weakness in the lower limbs before the age of 10 years, progression to upper limbs by the age of 12 years, and onset of visual loss in their 40s. The disease progressed, and at the age of 79 years, case F1‐II‐5 required the assistance of 2 people for sit‐to‐stand, mobilizing with the assistance of 2 plus Zimmer frames for transfers and short distances, and using a wheelchair for all other activities. Neurogenic‐type pain, particularly in the lower limbs, was difficult to control despite multiple lines of symptomatic treatment. Case F1‐II‐6 (age = 74 years) could only walk short distances with assistance from 1 individual. Neurological examination showed bilateral distal wasting from mid forearm and below the knee, with wasting of the thenar, hypothenar, and intrinsic muscle of the hands and distal lower limb muscles, pes cavus, and absent reflexes throughout in both patients (see Fig 1B). The MRC muscle score was 44 (of 60) in cases F1‐II‐5 and F1‐II‐6. Strikingly, both patients had severely reduced sensation in all limbs and in all modalities, with profound joint position sense loss up to the knee and vibration loss up to the iliac crest in both cases (see Table 1 and Supplementary Table 1). Vision was reduced (including color vision) and fundoscopy confirmed bilateral optic atrophy in both cases (see Fig 1C). The remaining cranial nerve examination was normal. The third sibling F1‐II‐9 (now deceased) was similarly affected.

Family 2 is of Scottish (paternal) and Italian (maternal) origin with 2 affected sisters (Patient F2‐II‐1, currently 31 years old; Patient F2‐II‐2, currently 29 years old) who presented with childhood onset of a mild decrease in balance, which progressed to difficulty with running in adolescence, and then to mild distal hand weakness in the 3rd decade. Both sisters ambulate independently without aids, and neither sister has symptoms of visual loss at their present ages. Neurological examination showed mild symmetrical distal wasting in hand intrinsic muscles and prominent distal lower limb muscle wasting with pes cavus and mild hammertoes. There was mild distal weakness in hand intrinsic muscles, in the upper limbs, and in the toes and distal lower legs and feet. The MRC scores were 56 (F2‐II‐1) and 54 (F2‐II‐2). Both sisters were areflexic, with reduced pinprick sense in the lower extremities and decreased vibration sense to the knees (see Table 1 and Supplementary Table 1). Color vision was reduced. Fundoscopy confirmed bilateral optic atrophy in both cases; the remainder of the cranial nerve examination was normal.

Nerve conduction studies (NCS) in all affected cases revealed a progressive, length‐dependent, sensorimotor, predominantly axonal neuropathy (Supplementary Table 2). Early in the disease (as seen in Family 2), the NCS show a predominantly axonal neuropathy affecting mainly the lower limbs with absent sensory responses. Mild conduction slowing with normal motor responses was recorded in the upper limbs. The natural history of this disorder in the older individuals from Family 1 (F1‐II‐5 and F1‐II‐6) with data recorded over 20 years of disease duration suggests that untreated, the neuropathy progresses in a length‐dependent fashion and is characterized by prolonged distal motor latency (DML), severe reduction of compound muscle action potentials (CMAPs), and absent sensory responses affecting upper and lower limbs, as confirmed on NCS. Interestingly, both patients present with significantly more prolonged DML in the median compared to ulnar nerve (6.1 vs 3.6 milliseconds). Although it is possible that this is secondary to a longstanding median compression lesion at the wrist, we cannot exclude that this finding is disease‐related. The reduced CMAPs were associated with reduction of conduction velocities in the intermediate range mainly in the median nerves. Electromyography in all cases tested was consistent with chronic denervation with no myopathic changes.

To further ascertain the exact type of neuropathy, we performed a nerve biopsy in case F1‐II‐6. It showed diffuse and severe depletion of both small and large myelinated axons with regenerating clusters but no “onion bulbs.” This confirms a longstanding axonopathy with no overt demyelination (see Fig 1D).

All cases reported here had pale optic discs on clinical examination. We performed visual evoked potentials (VEPs) to further describe the involvement of the optic nerve in the course of disease. We found that VEPs in Case F1‐II‐6 had decreased considerably in amplitude from 7.9 and 7.4 in each eye to 1.8 and 1.9 over a 20‐year period, indicating axonal loss and bilateral visual pathway involvement (see Supplementary Table 2). Similar results were confirmed in case F1‐II‐5. Family 2 has not been investigated with VEPs.

### *Identification of Disease‐Causing* PDXK *Variants*

Previous extensive genetic,^37^ metabolic, and mitochondrial investigations failed to identify any acquired or known causes of inherited polyneuropathy in all cases. WGS and homozygosity mapping in Family 1 revealed a shared homozygous region from 42 to 45Mb on chromosome 21. This region included a shared homozygous variant in the 2 affected siblings that was carried by unaffected family members in heterozygous state: *PDXK* (NM_003681) c.682G>A (p.Ala228Thr). This variant was absent in 150 Cypriot controls. The p.Ala228Thr is absent from thegnomAD database in homozygous state though reported in 7 heterozygous of atotal of 250586 alleles (total allele frequency of 0.00002793).^23^

Independently, homozygosity mapping in Family 2 revealed a 1.35Mb region of homozygosity on chromosome 21. WES in Family 2 identified a shared homozygous variant in this region, the c.659G>A p.(Arg220Gln) in the 2 affected sisters, carried by each parent. This variant is absent from gnomAD in homozygous state and reported in 5 heterozygous European alleles from 250146 (total allele frequency of 0.00001999).^23^ It is predicted to be deleterious to protein function by *in silico* programs (Combined Annotation Dependent Depletion score = 24). Sanger sequencing in affected individuals and carriers confirmed both variants (see Fig 1E).

*PDXK* is ubiquitously expressed in humans encoding for PL kinase (PDXK), a cytoplasmic protein that converts vitamin B_6_ to its active form, PLP (see Fig 1F). PLP is an essential cofactor for >70 human enzymes representing diverse, essential biological pathways including amino acid and neurotransmitter metabolism.

**Figure 2:**
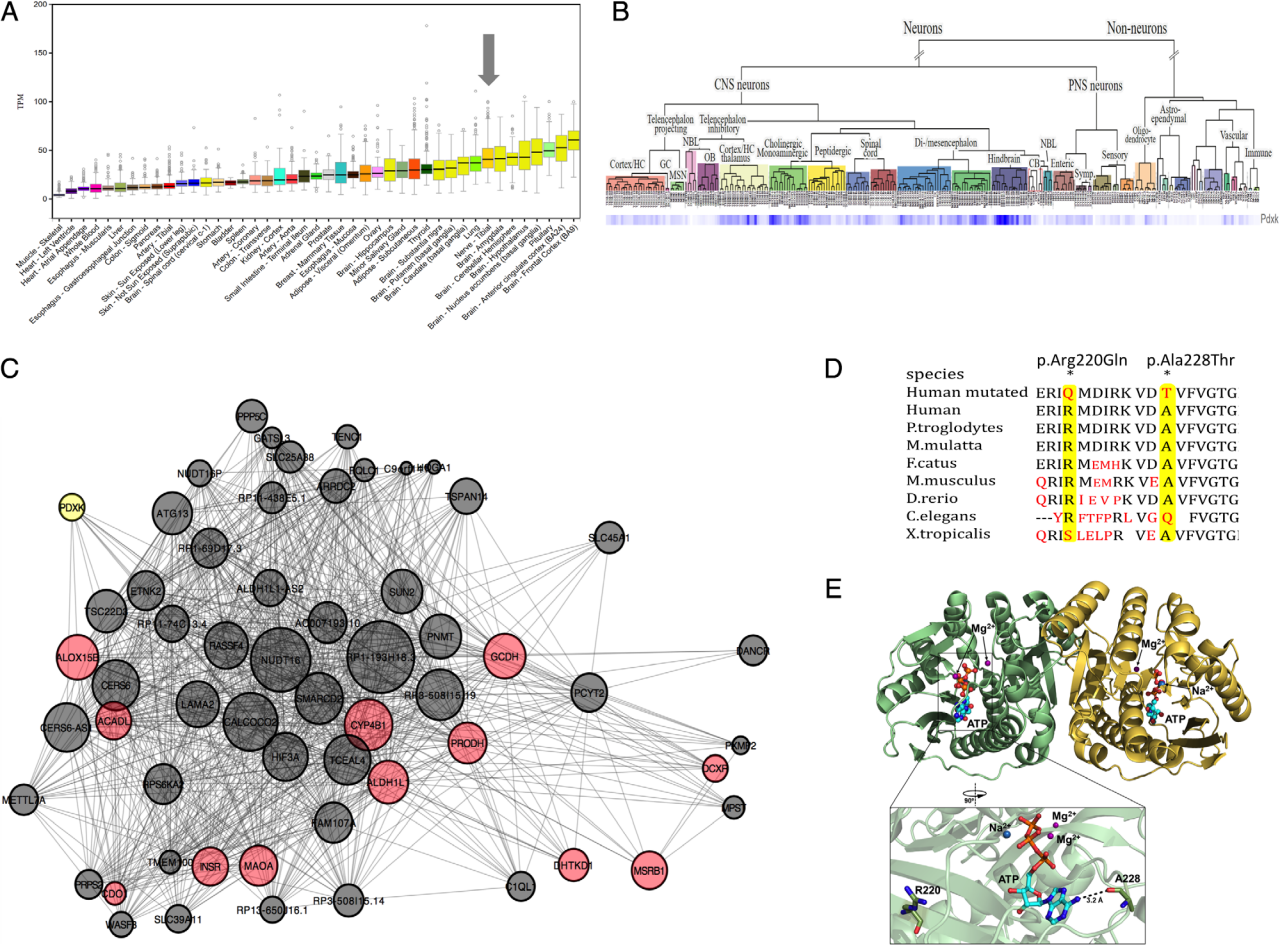
*PDXK* is highly expressed in the peripheral and central nervous systems and in the same regulon with genes already linked to axonal peripheral neuropathies. (A) Expression of *PDXK* in human tissues. Box and whisker plots show the expression of *PDXK* across multiple human tissues. Data were generated by the GTEx Consortium. Expression in tibial nerve is highlighted with a dark gray arrow and is among the tissues with the highest *PDXK* expression. TPM = Transcripts Per Kilobase Million. (B) Expression of *PDXK* in multiple cell types of the mouse central nervous system (CNS) and peripheral nervous system (PNS) generated using single cell RNA‐seq. *PDXK* gene expression across single cells isolated from the mouse central and peripheral nervous systems and displayed using a heatmap demonstrates highest expression of this gene in neurons of the mouse hindbrain,^38^ with expression in the peripheral neurons including sensory neurons. CB = cerebellum; GC = glial cells; HC = hippocampus; MSN = medium spiny neurons; NBL = neuroblasts; OB = olphactory bulb; Symp = sympathetic. (C) Top‐down plot of the black module genes in the tibial nerve tissue. Only the most connected genes are shown. *PDXK* gene is highlighted in yellow. Genes known to be associated with the Gene Ontology term GO:0055114, oxidation–reduction process, are highlighted in red. The size of gene nodes reflects their connectivity with the rest of the genes in the module. *PDXK* is among the top 60 most connected genes. Proximity of genes in the plot reflects their similarity in terms of shared connections with other genes. Interestingly, within the *PDXK* regulon from the tibial nerve, we found *DHTKD1* already linked to Mendelian disorders and associated with primary peripheral axonal neuropathy.^59^, ^60^ (D) Conservation of p. Ala228Thr and p.Arg220Gln in *PDXK* across species. (E) Crystal structure of human pyridoxal kinase with bound adenosine triphosphate (ATP; PDB accession number 3KEU). PDXK is a dimeric enzyme with 1 active site per monomer^65^ (monomers A and B are depicted in green and yellow, respectively). In the PDXK structure, the backbone‐carbonyl oxygen of alanine 228 establishes a hydrogen bond with the adenine NH_2_ group of ATP. The active site of each monomer binds 1 ATP molecule, two Mg^2+^ ions, and 1 Na^2+^ ion. The ATP‐binding site is composed of a β‐loop‐β structure, often referred as a flap, which provides numerous hydrogen‐bond interactions to the ATP β‐ and γ‐phosphates, and sequesters the ATP for catalysis.^39^ Arginine 220 is located in the β9, in the vicinity of the ATP‐binding site. PDB = Protein Data Bank.

Because *PDXK* mutations produce a peripheral rather than a predominantly CNS disease, we investigated these differences further *in silico* using public bulk tissue^25^ and cell‐specific transcriptomic data.^38^ This demonstrated that human peripheral (tibial–mixed motor and sensory) nerve is among the tissues with the highest expression of *PDXK* (Fig 2A), with evidence for expression specifically in peripheral sensory neurons, based on single cell mouse transcriptomic data (see Fig 2B).^38^ This showed different co‐expression profiles in brain compared to peripheral nerve tissue. Weighted gene co‐expression analysis demonstrated that in 9 of 11 brain regions analyzed, *PDXK* was coexpressed with genes relating to synaptic transmission and neuronal identity, whereas co‐expression data from the tibial nerve showed that *PDXK* was located within a module enriched for genes involved in oxidation‐reduction processes (GO: 055114; false discovery rate–corrected *p* = 2.36 × 10^−9^; see Fig 2C and Supplementary Table 3).

### *Assessment of the Impact of* PDXK *Variants on Protein Structure and Enzymatic Activity*

Arginine 220 and alanine 228 are evolutionarily conserved amino acids in eutherians (see Fig 2D). Arginine 220 is located in the β9 in the vicinity of the ATP‐binding site.^39^ Alanine 228 is located in the ATP‐binding pocket of PL kinase (see Fig 2E), a critical region of the kinase, part of the loop region between strand β11 and helix α7 that rotates away from the active site, allowing binding of the ATP adenine.^39^ Although the Ala228 side‐chain does not contact ATP directly, its backbone is involved in recruiting ATP to the catalytic pocket.

**Figure 3:**
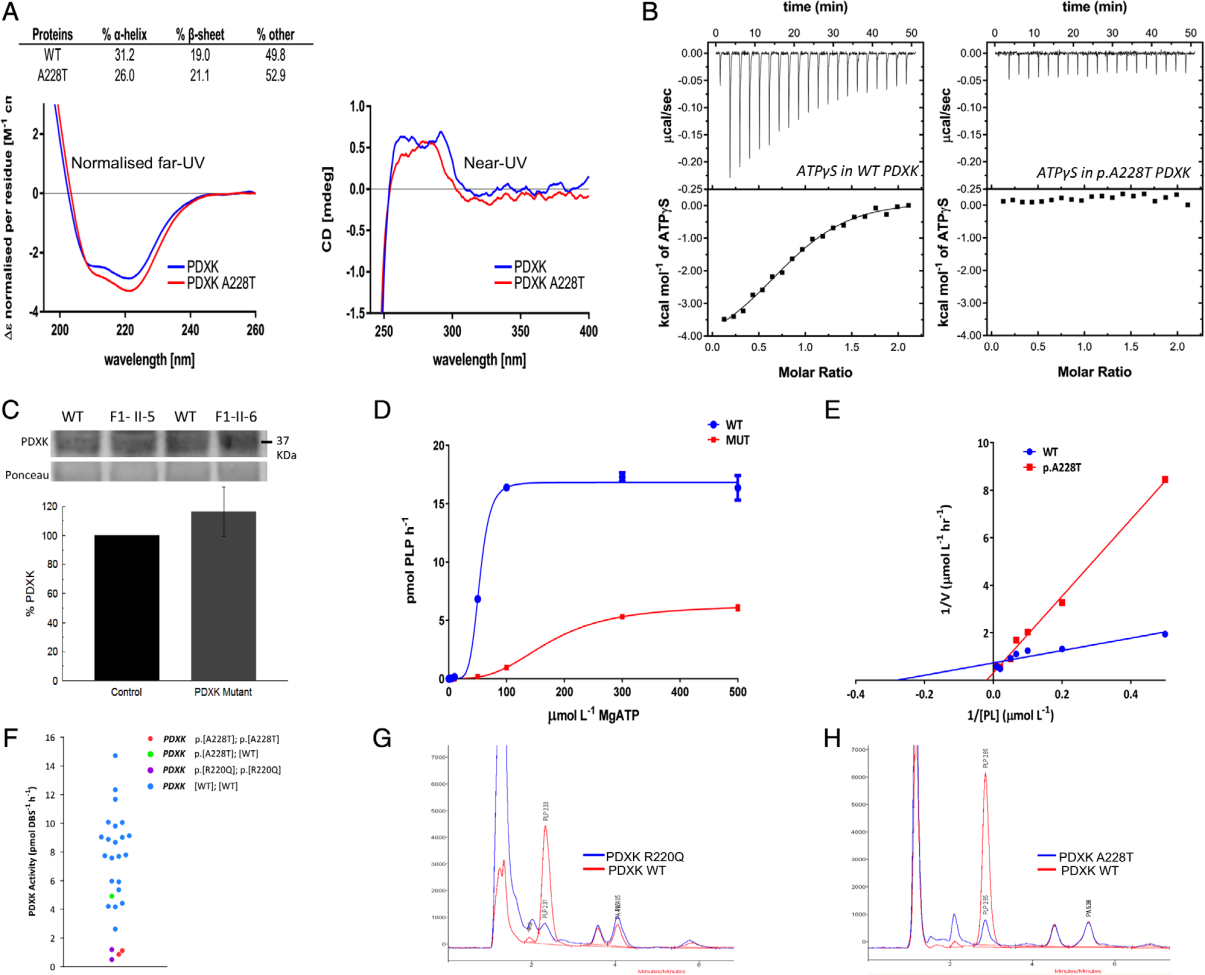
*PDXK* mutations lead to reduced pyridoxal (PL) kinase enzymatic activity and low PL 5′‐phosphate (PLP). (A) Circular dichroism analyses of recombinant PDXK wild‐type (WT) and p.Ala228Thr mutant proteins. The left and right panels show the normalized far‐ultraviolet (UV) and near‐UV spectra of the 2 proteins, respectively. A clear difference in secondary structure content between the 2 proteins is observed from the far‐UV experiment. CD = circular dichroism. (B) Analysis of the interaction of nonhydrolyzable analogue adenosine 5′‐(3‐thiotriphosphate) tetralithium salt (ATPγS) with PDXK WT and p.Ala228Thr mutant proteins by isothermal titration calorimetry. The left panel shows the titration of ATPγS (250μM) into a PDXK WT solution (25μM). The thermogram shows that the interaction was entropically and enthalpically favored, with ΔH = −3.27 ± 0.42kcal/mol, TΔS = −4.42 ± 0.48kcal/mol, K_D_ = 2.33 ± 0.25μM, and ΔG = −7.69 ± 0.06kcal/mol. The stoichiometry was 0.80 ± 0.02μM, indicating that each molecule of PDXK binds to 1 molecule of ATPγS. The right panel reports the titration of ATPγS (250μM) into a PDXK p.Ala228Thr solution (25μM). The experiment showed no interaction under the experimental conditions tested, suggesting that the mutation affected the ability of the kinase to bind the analogue substrate ATPγS. (C) Western blot analysis shows normal expression of the PDXK protein in cases compared to controls. (D) Activity of recombinant WT and p.Ala228Thr PL kinase protein measured as PLP formation. Conditions: 0–100μmol/l PL, 300μmol/l MgATP, 20mmol/l potassium phosphate, pH 7.0, 37°C, 10‐minute incubation with 100ng recombinant protein. Points displayed are a mean of 3 repeats. V_max_: WT = 2.17μmol/l/h, p.Ala228Thr = 2.52μmol/l/h. K_m_: WT = 14.53μmol/l, p.Ala228Thr = 31.93μmol/l. MUT = mutant. (E) Kinetics of recombinant WT and p.Ala228Thr PL kinase protein upon variation of PL concentration. PL kinase activity of recombinant human WT and p.Ala228Thr PDXK protein is measured as PLP formed after incubation with the substrate PL. Incubations were performed in the presence of variable concentrations of MgATP (0–500μmol/l) and 50μmol/l PL. Kinetics were sigmoidal, and parameters established were as follows. WT: k_0.5_ = 53.4μmol/l, V_max_ = 16.8pmol/h; p.Ala228Thr: k_0.5_ = 174.4μmol/l, V_max_ = 6.3pmol/h. Results indicate a dramatic reduction in the catalytic efficiency of the p.Ala228Thr PDXK protein. n = 3 at each data point. (F) Erythrocyte PDXK activity in dried blood spots (DBSs) from cases homozygous for the p.Ala228Thr and p.Arg220Gln versus controls (age = 15–92 years). Patients homozygous for p.Ala228Thr and p.Arg220Gln have lower activity than all controls. Activity measured as PLP formed after incubation of a 3mm DBS punch with PL. Each sample was analyzed in duplicate, and the mean is shown. There was no correlation of PDXK activity with age. (G, H) Comparison of plasma PLP concentrations (retention time = 2.78/2.84 minutes) in control (red) and cases carrying the *PDXK* mutation (blue) p.Arg220Gln (G) and p.Ala228Thr (H) show a significant reduction of PLP in the case samples (7.8 and 9nmol/l, respectively) versus control (control range = 25–75nmol/l).

Far‐UV CD spectra analysis of both WT and p.Ala228Thr revealed that, whereas both proteins were folded, there were small but significant differences in their secondary structure (Fig 3A). WT PDXK had an estimated α‐helix and β‐strand content of 31.2% and 19%, respectively, similar to previous X‐ray studies (PDB accession number 3KEU).^40^ In the mutant protein, 26.0% and 21.1% of helical and strand content, respectively, were estimated, suggesting altered protein conformation. Furthermore, near‐UV CD analysis revealed distinctive CD fingerprints for WT and p.Ala228Thr proteins, indicating conformational rearrangements of aromatic side‐chains^41^ around the catalytic pocket, most likely hindering the enzyme's ability to bind ATP.

We performed ITC confirming that p.Ala228Thr affected the ATP‐binding ability of PDXK. Interaction of WT PDXK with the nonhydrolyzable ATP analogue ATPγS generated well‐interpolated, sigmoid‐shaped curves, based on an independent and equivalent binding site model centered on 1:1 stoichiometry. By contrast, no association between p.Ala228Thr and ATPγS was observed (see Fig 3B).

Whereas no reduction in the expression of the mutant protein (p.Ala228Thr) compared to WT PDXK in case‐derived fibroblasts was evident (see Fig 3C), kinetic studies with human recombinant PDXK showed an approximate 2‐fold increase in the K_m_ of the p.Ala228Thr mutant protein (31.9μmol/l) for PL compared to WT protein (14.5μmol/l) and a reduction in the V_max_ of the mutant protein (p.Ala228Thr) to 0.95μmol/l/h compared to 2.52μmol/l/h for WT PDXK. Upon variation of MgATP concentration, sigmoidal kinetics were observed (WT: k_0.5_ = 53.4μmol/l, V_max_ = 16.8pmol/h; p.Ala228Thr: k_0.5_ = 174.4μmol/l, V_max_ = 6.3pmol/h), indicating cooperative binding between the 2 substrates, ATP and PL (see Fig 3D, E).

Analysis of erythrocyte PDKX activity from cases carrying the p.Arg220Gln and p.Ala228Thr substitutions further confirmed the deleterious effect of these variants on PL kinase activity (see Fig 3F). The PDKX activity in the 4 affected cases was reduced (1.1 and 0.8pmol DBS^−1^h^−1^ in Family 1 and 1.19 and 0.5pmol DBS^−1^h^−1^ in Family 2) relative to controls (2.6–14.7pmol DBS^−1^h^−1^, mean = 8.0). Heterozygote activity (n = 1, F‐1‐III‐1) was within normal range (4.9pmol DBS^−1^h^−1^). No correlation of DBS PL kinase activity with age was identified in controls. When segregated according to gender, a difference was identified between control males and females (9 males: mean = 9.2, range = 5.9–14.7; 12 females: mean = 7.1, range = 2.6–11.7) that approached significance (*p* = 0.1099 by untailed *t* test). An insertion located in the erythroid‐specific promoter region of PL kinase has been associated with reduced erythrocyte PDXK activity.^21^ Sequencing this region showed that the reduced activity of all affected individuals was not attributable to this insertion (Supplementary Table 4).^42^

Finally, we measured plasma PLP concentrations in all affected individuals. Plasma PLP was greatly reduced in cases carrying p.Arg220Gln and p.Ala228Thr compared to age‐matched controls (see Fig 3G, H).

To quantify the axonal damage, we measured the NFL protein in plasma from patients carrying *PDXK* mutations. NFL, a major axonal cytoskeletal protein, is released into cerebrospinal fluid and blood during axonal breakdown,^43^ providing a dynamic biomarker of axonal damage in neurodegenerative disorders, including inherited neuropathies.^44^ Both p.Arg220Gln and p.Ala228Thr variants were associated with high NFL (Case F2‐II‐1, 49.6pg/ml; Case F1‐II‐5, 25pg/ml), within the range of NFL concentrations published for other axonal CMTs.^44^

### *Supplementation with PLP Is Associated with Biochemical Correction and Clinical Improvement*

Based on clear pathogenicity of the *PDXK* mutations leading to low PLP, Case F1‐II‐5 and F1‐II‐6 were treated with oral PLP. High doses of pyridoxine are known to be neurotoxic^45^; therefore, we used PLP, which is hydrolyzed to PL, to increase the upstream PL and force the flux with an enzyme with a high K_m_ based on our results with recombinant p.Ala228Thr protein kinetics. We initiated PLP replacement with 50mg/day PLP, a dose that was commonly used in adults taking PLP as a vitamin supplement with no reported side effects. We aimed to achieve plasma PLP concentration above the normal range by PLP replacement, and this was achieved with 50mg/day.

**Figure 4:**
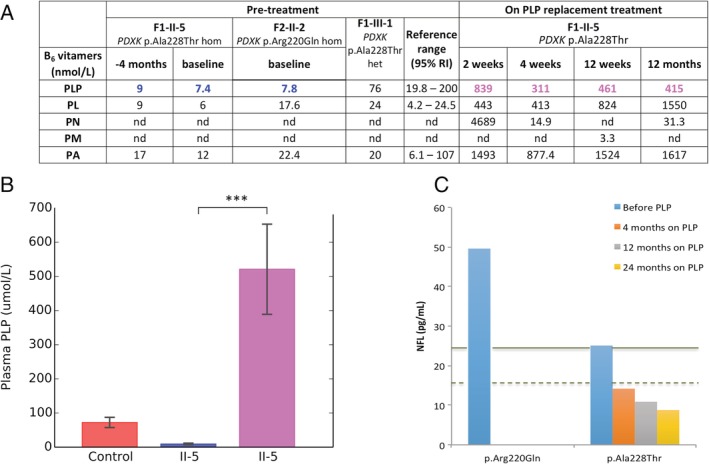
Pyridoxal (PL) 5′‐phosphate (PLP) supplementation in patients with *PDXK* mutations can rescue the biochemical phenotype. (A) Concentrations of plasma B_6_ vitamers in affected homozygous (hom) cases for p.Ala228Thr (F1‐II‐5), p.Arg220Gln (F2‐II‐2), and a heterozygous (het; F1‐III‐1) *PDXK* mutation carrier. The levels prior to supplementation were compared to the published range of B_6_ vitamers in adult controls (n = 523)^66^ not receiving PLP. All units are nmol/l, except for PNP, which is given in concentration units. nd = not detected; PA = 4‐pyridoxic acid; PM = pyridoxamine; PN = pyridoxine; RI = reference interval. (B) The effect of PLP supplementation on plasma PLP concentrations in a case with *PDXK* mutations. The red bar represents the PLP levels in a group of adult controls with no B_6_ supplementation. There is a significant difference in the plasma PLP concentration of F1‐II‐5 before supplementation *(blue bar)* and on PLP replacement (*magenta bar*; ***p < 0.05). The difference between groups was tested with the use of a 1‐way analysis of variance test followed by the Tukey–Kramer test. The horizontal lines on the bars indicate mean value ±1 standard deviation. (C) Neurofilament light chain (NFL) concentrations in plasma from cases with homozygous p.Arg220Gln and p.Ala228Thr *PDXK* mutations and a heterozygous carrier (F1‐III‐1). The blue bars show that NFL levels prior to PLP supplementation are high and consistent with values published in other inherited peripheral neuropathies *(solid line)*,^44^ indicating ongoing axonal damage. The orange, gray, and yellow bars show the NFL levels in the cases from Family 1 at 4, 12, and 24 months on PLP supplementation, respectively. The levels have reduced to that of normal controls *(dashed line)* and continued to improve with longitudinal follow‐up, suggesting an amelioration of the axonal breakdown.

Two weeks after initiating the supplementation, plasma PLP levels had increased considerably without any side effects. PLP levels remained stable at 1, 3, 12, 18, and 24 months on PLP replacement (Fig 4A, B, Table 2), with improvement in symptoms (Supplementary Table 5). Clinical improvement was noted after the first 3 months of PLP replacement in the absence of any additional physiotherapy. Sensory examination and vision remained unchanged in both cases. Main motor improvements were in elbow and wrist flexion/extension and hip and knee flexion/extension, with modest improvement in finger extension (see Supplementary Table 1), leading to 8‐ and 6‐point improvement in the MRC sum score at 18 months on PLP replacement in Case F1‐II‐5 and Case F1‐II‐6, respectively. At last examination, 24 months on PLP replacement, patients were ambulating with high‐stepping gait, unaided (Supplementary Video). Furthermore, neuropathic pain has completely subsided; neither case requires additional analgesic medication.

**Table 2:** Plasma B_6_ Vitamer Profiles for Patients with PROSC, PNPO, and PDXK Deficiency Supplemented with PLP

	Age	B_6_ (dose)	PLP	PL	PA	PN	PNP	PMP	PM
Control range^a^	4.3–16 years	None	46–321	5–18	16–139	nd–0.6	nd	nd–9	nd
PROSC^b^	3 years	PLP (70mg QDS)	2,769^c^	796^c^	2,043^c^	0.5	nd	nd	nd
PROSC^b^	6 months	PLP (45mg/kg/day)	2,166^c^	1,695^c^	700^c^	nd	nd	nd	nd
PNPO^d^	2 years	PLP (30mg/kg/d)	580^c^	427^c^	793^c^	575^c^	43^c^	18^c^	193^c^
PNPO^d^	10 years	PLP (30mg/kg/d)	633^c^	5,798^c^	7,926^c^	599^c^	77^c^	101^c^	2,731^c^
PDXK^e^	79 years	PLP (50mg/d)	415^c^	1,550^c^	1,617^c^	31^c^	nd	nd	nd

All units are given as nmol/L, except for PNP, which is given in concentration units.

a Control range as described in Footitt et al^46^.

b Data from Darin et al^47^.

c Values outside the reference range.

d Data from Footitt et al 2013. PLP doses received by PROSC and PNPO patients were larger than the doses given to the pyruvate kinase–deficient patients (II‐5 and II‐6).

e Values at 12 months on treatment with PLP.

nd = not detected; PA = 4‐pyridoxic acid; PL = pyridoxal; PLP = pyridoxal 5′‐phosphate; PM = pyridoxamine; PMP = pyridoxamine 5′‐phosphate; PN = pyridoxine; PNP = pyridoxine 5′‐phosphate; QDS = 4 times per day.

These results led to an improvement of 5 points in the NIS score in Case F1‐II‐5 and 4 points in Case F1‐II‐6 based on motor, pain, and fatigue subsets. Similarly, the CMT neuropathy score on PLP replacement improved by 4 points and 2 points in Cases F1‐II‐5 and F1‐II‐6, respectively (see Supplementary Table 5).

Electrophysiological studies performed 18 months after PLP supplementation have demonstrated no disease progression, with no significant changes in the motor and sensory responses. Whereas the distal motor responses were not significantly different from previous studies, the electromyogram of the proximal muscles showed motor unit recruitment and interference pattern improvement in the proximal muscles (quadriceps and tibialis anterior muscles, biceps and flexor carpi radialis) with activity present in the tibialis anterior that was unrecordable on previous studies.

The clinical response to PLP replacement was further supported by measuring NFL protein in plasma before and after PLP replacement from the *PDXK* cases in Family 1. A reduction of NFL levels to the normal control range^44^ (median = 13pg/ml, range = 11–18pg/ml; see Fig 4C) occurred upon restoration of PLP levels and continued to improve with PLP supplementation. At 24 months with PLP supplementation, the NFL level was 2.5 times lower than before supplementation.

## Discussion

Here, we show that biallelic mutations in *PDXK* lead to peripheral neuropathy with optic atrophy. We have shown previously that several inherited disorders affecting B_6_ vitamer metabolism, or resulting in inactivation of PLP, are implicated in neurological conditions. These include PNPO (Mendelian Inheritance in Man [MIM]: 603287),^48^ ALDH7A1 (MIM: 266100),^49^, ^50^ and PROSC deficiency (MIM: 604436),^51^, ^52^ where the lack of PLP in the brain leads to early onset, vitamin B_6_‐dependent epilepsy refractory to anticonvulsants. In most cases, delayed vitamin B_6_ supplementation results in early death or severe handicap in these disorders. The PDXK activity is tissue‐specific under PLP‐deficiency conditions. In rats, B_6_ deficiency leads to rapid decreases in PDXK activity in the peripheries (liver, muscle, and plasma), with maintained PDXK activity and B_6_ supply in the brain.^53^, ^54^ This could explain the absence of seizures in humans with PDXK deficiency who present predominantly with peripheral nerve disease and normal CNS function. Furthermore, our *PDXK* co‐expression network analyses from peripheral nerve suggest a link to a module enriched for genes involved in oxidation‐reduction processes in which mitochondria play a significant role. PLP is a cofactor for several mitochondrial enzymes, but it is not certain how PLP is transported into mitochondria in humans; in yeast, there is evidence for involvement of Mtm1p.^55^ Although we acknowledge that coexpression networks generated from bulk RNA‐seq of peripheral nerves are complex to interpret, there is growing evidence for the presence and importance of axonal RNA transport.^56^, ^57^, ^58^ Thus, our findings are interesting given the strong phenotypic similarity (optic atrophy and axonal polyneuropathy) to other disorders caused by mutations in genes involved in mitochondrial functions associated with neurodegeneration, such as *OPA1* (MIM: 605290), *DHTKD1* (MIM: 614984), *MFN1* (MIM: 608506), and *MFN2* (MIM: 608507). Interestingly, within the *PDXK* regulon from the tibial nerve, we found *DHTKD1* already linked to Mendelian disorders and associated with primary peripheral axonal neuropathy.^59^, ^60^ This suggests that within this regulon there may be other genes involved in neuronal maintenance that could cause a similar disease phenotype to be discovered.

Clinically, the *PDXK*‐related neuropathy presented with mixed sensory and motor involvement in early childhood and was associated with optic atrophy later in adulthood. Electrophysiologically, one of the earliest signs of the disease was the absence of sensory responses even when the motor responses were within normal limits, as seen in Family 2. We note that the patients with longstanding disease (Family 1) had significant standing and walking difficulties despite a mild proximal motor deficit most likely due to the combination of severe distal motor involvement with severe proprioceptive impairment, extending to the proximal joints, exacerbated by the severely impaired vision.^61^

Based on our collective experience in rare diseases, we acknowledge that it is difficult to implement randomized case–control studies for these patients and the subsequent limitations when designing therapeutic interventions. We note that some clinical improvements seen in the patients reported here, such as pain and fatigue, are susceptible to placebo effects. Furthermore, the severity of the polyneuropathy in the cases that received PLP supplementation and the absence of more proximal nerve conduction studies are limitations for the characterization of the neurophysiological phenotype and response to treatment. However, although we are limited in our ability to fully explain the mechanism of clinical improvement, the supplementation with PLP was associated with normalization of the biochemical profile and improved clinical outcomes in individuals F1‐II‐5 and F1‐II‐6. The clinical improvement was supported by the normalization of PLP levels, stable electrophysiological picture, and NFL normalization after treatment. Furthermore, animal models of *PDXK* knockdown, B_6_ dietary deficiency, and pharmacological models all showed significant decreases in circulating PLP levels, with reversible adverse effect(s) on the peripheral/sensory nerve similar to the human phenotype observed here (Table 3). The severe sensory involvement recorded at least 20 years before treatment most likely accounted for the absence of a clinical effect on the sensory function in the 2 patients from Family 1. We are limited in our ability to fully quantify the potential for electrophysiological improvement at this stage in the disease due to severe axonal loss, the short interval of PLP replacement, and the absence of more proximal nerve conduction studies. Therefore, larger studies with treatment started earlier in the disease are warranted to assess the full potential benefit from treatment.

**Table 3:** Vitamin B_6_–Related Disease Models

Model	Phenotype	Mechanism	Effect on B_6_ Pathway	Response to PLP Supplementation
Pharmacological models				
Isoniazid, L‐dopa, gentamicin, D‐penicillamine	Axonal peripheral neuropathy^1^	Interaction between the reactive aldehyde group of PLP, with nucleophilic acceptors such as amine, hydrazine, hydroxylamine, or sulfydryl groups rendering PLP inactive	Decrease in serum PLP	Prevents onset of peripheral neuropathy
Methylxanthines	Axonal peripheral neuropathy^2^, ^3^	Directly inhibit enzymes involved in B_6_ metabolism		
Carbamazepine, vigabatrin, sodium valproate	Axonal peripheral neuropathy^4^, ^5^, ^6^, ^7^	Potent hepatic inducers of cytochrome P450, induce enzymes involved in the catabolism of PLP		
B_6_ antivitamin ginkgotoxin (4′‐O‐methylpyridoxine)	Epileptic convulsions, leg paralysis, and loss of consciousness^8^	Analogue of PLP; inhibition of pyridoxal kinase by serving as an alternate substrate for the enzyme	Decrease in PLP formation	NA
Animal models				
Mouse	Preweaning lethality, MGI: 1351869	*PDXK* knockout	NA	NA
Mouse	Abnormal walking track patterns, axonal peripheral neuropathy with intact myelin on nerve biopsy^9^	PLP dietary‐deficient	Decrease in serum PLP	Complete reversal of symptoms with PLP supplementation
Rat	Tissue‐specific PDXK response to B_6_ deficiency^10^, ^11^	B_6_ diet deficient	PDXK activity rapidly decreases in the liver, muscle, and plasma compared to the brain	Reversal with PLP supplementation
*Caenorhabditis elegans*	Sensory–motor integration deficit in neuromuscular behavior^12^	*PDXK* knockout	NA	Rescued by expression of a WT transgene
*Drosophila*	Motility and eye dysfunction with compromised climbing ability, ommatidial array disruption, and decreased longevity^13^	*PDXK* knockdown by RNA interference	NA	Rescued by expression of a WT transgene

PLP = pyridoxal 5′‐phosphate; MGI = Mouse Genome Informatics; NA = not applicable; WT = wild‐type.

### *Conclusions*

We show that biallelic mutations in *PDXK* cause autosomal recessive axonal peripheral polyneuropathy leading to disease via reduced PDXK enzymatic activity and low PLP. We show that the biochemical profile in affected individuals can be rescued with PLP supplementation and that this is associated with improvement in clinical scales. Therefore, we recommend that *PDXK* mutations should be screened for in patients with autosomal recessive early onset polyneuropathy, and supplementation with PLP should be started promptly with long‐term monitoring of clinical outcomes. Furthermore, B_6_ vitamers can be linked to diseases through a wide range of processes and genes involved in the vitamin B_6_ pathway. Therefore, our results can be extended to other neuropathies of different etiology characterized by reduction of PLP levels or reduction of PLP‐dependent enzyme activities. Collectively, these data expand the genetic causes of primary polyneuropathy and the heterogeneity of inborn errors of vitamin B_6_ metabolism and identify PLP as a potential therapeutic target for this disorder.

## Author Contributions

V.C., H.H., N.W.W., J.E.R., P.T.C., P.B.M., K.M.B., and J.W.C. contributed to conception and design of the study. All authors contributed to acquisition and analysis of data. V.C., H.H., P.T.C., P.B.M., M.P.W., M.R.C., G.A., K.M.B., and J.W.C. contributed to drafting the text and preparing the figures.

## Potential Conflicts of Interest

Nothing to report.

## Supporting information


**Supplementary Table 1** Extended neurological examination pre and on PLP supplementation in patients with *PDXK* mutations.
**Supplementary Table 2.** Electrophysiological studies in patients with *PDXK* mutations.
**Supplementary Table 3. Gene co‐expression network analysis for *PDXK*.**
**Supplementary Table 4. Description of the genotype of promoter region for the control group and *PDXK* p.Ala228Thr cases.**
**Supplementary Table 5.** Clinical response to pyridoxal 5’‐phosphate (PLP) treatment.
**Supplementary Table 6.** SYNaPS Study Group collaborators.

**Supplementary Video.** Clinical phenotype of cases homozygous for the p.Ala228Thr *PDXK* mutation was rescued by PLP supplementation. Improvement in power, pain and fatigue contributed to patients regaining their ability to ambulate with a high‐stepping gait, unaided.

## References

[1] Gess B , Baets J , De Jonghe P , et al. Ascorbic acid for the treatment of Charcot‐Marie‐Tooth disease. Cochrane Database Syst Rev 2015;(12):CD011952. 10.1002/14651858.CD011952 PMC6823270 26662471

[2] d'Ydewalle C , Benoy V , Van Den Bosch L . Charcot‐Marie‐Tooth disease: emerging mechanisms and therapies. Int J Biochem Cell Biol 2012;44:1299–1304. 22575637 10.1016/j.biocel.2012.04.020

[3] Züchner S , Vance JM . Mechanisms of disease: a molecular genetic update on hereditary axonal neuropathies. Nat Clin Pract Neurol 2006;2:45–53. 16932520 10.1038/ncpneuro0071

[4] Braathen GJ , Sand JC , Lobato A , et al. Genetic epidemiology of Charcot‐Marie‐Tooth in the general population. Eur J Neurol 2011;18:39–48. 20482598 10.1111/j.1468-1331.2010.03037.x

[5] Skre H . Genetic and clinical aspects of Charcot‐Marie‐Tooth's disease. Clin Genet 1974;6:98–118. 4430158 10.1111/j.1399-0004.1974.tb00638.x

[6] Nicolaou P , Zamba‐Papanicolaou E , Koutsou P , et al. Charcot‐Marie‐Tooth disease in Cyprus: epidemiological, clinical and genetic characteristics. Neuroepidemiology 2010;35:171–177. 20571287 10.1159/000314351

[7] Nelis E , Van Broeckhoven C , De Jonghe P , et al. Estimation of the mutation frequencies in Charcot‐Marie‐Tooth disease type 1 and hereditary neuropathy with liability to pressure palsies: a European collaborative study. Eur J Hum Genet 1996;4:25–33. 8800924 10.1159/000472166

[8] Morocutti C , Colazza GB , Soldati G , et al. Charcot‐Marie‐Tooth disease in Molise, a central‐southern region of Italy: an epidemiological study. Neuroepidemiology 2002;21:241–245. 12207152 10.1159/000065642

[9] Murphy SM , Laura M , Fawcett K , et al. Charcot‐Marie‐Tooth disease: frequency of genetic subtypes and guidelines for genetic testing. J Neurol Neurosurg Psychiatry 2012;83:706–710. 22577229 10.1136/jnnp-2012-302451 PMC3736805

[10] Skinner A , Turner‐Stokes L . The use of standardized outcome measures in rehabilitation centres in the UK. Clin Rehabil 2006;20:609–615. 16894804 10.1191/0269215506cr981oa

[11] Shy ME , Blake J , Krajewski K , et al. Reliability and validity of the CMT neuropathy score as a measure of disability. Neurology 2005;64:1209–1214. 15824348 10.1212/01.WNL.0000156517.00615.A3

[12] Murphy SM , Herrmann DN , McDermott MP , et al. Reliability of the CMT neuropathy score (second version) in Charcot‐Marie‐Tooth disease. J Peripher Nerv Syst 2011;16:191–198. 22003934 10.1111/j.1529-8027.2011.00350.x PMC3754828

[13] Li H , Durbin R . Fast and accurate long‐read alignment with Burrows‐Wheeler transform. Bioinformatics 2010;26:589–595. 20080505 10.1093/bioinformatics/btp698 PMC2828108

[14] McKenna A , Hanna M , Banks E , et al. The Genome Analysis Toolkit: a MapReduce framework for analyzing next‐generation DNA sequencing data. Genome Res 2010;20:1297–1303. 20644199 10.1101/gr.107524.110 PMC2928508

[15] Van der Auwera GA , Carneiro MO , Hartl C , et al. From FastQ data to high confidence variant calls: the Genome Analysis Toolkit best practices pipeline. Curr Protoc Bioinformatics 2013;43:11.10.1–11.10.33. 25431634 10.1002/0471250953.bi1110s43 PMC4243306

[16] DePristo MA , Banks E , Poplin R , et al. A framework for variation discovery and genotyping using next‐generation DNA sequencing data. Nat Genet 2011;43:491–498. 21478889 10.1038/ng.806 PMC3083463

[17] Danecek P , McCarthy SA . BCFtools/csq: haplotype‐aware variant consequences. Bioinformatics 2017;33:2037–2039. 28205675 10.1093/bioinformatics/btx100 PMC5870570

[18] Yang H , Wang K . Genomic variant annotation and prioritization with ANNOVAR and wANNOVAR. Nat Protoc 2015;10:1556–1566. 26379229 10.1038/nprot.2015.105 PMC4718734

[19] Seelow D , Schuelke M , Hildebrandt F , Nürnberg P. HomozygosityMapper—an interactive approach to homozygosity mapping. Nucleic Acids Res 2009;37(Web Server issue):W593–W599. 19465395 10.1093/nar/gkp369 PMC2703915

[20] Narasimhan V , Danecek P , Scally A , et al. BCFtools/RoH: a hidden Markov model approach for detecting autozygosity from next‐generation sequencing data. Bioinformatics 2016;32:1749–1751. 26826718 10.1093/bioinformatics/btw044 PMC4892413

[21] 1000 Genomes Project Consortium , Auton A , Brooks LD , et al. A global reference for human genetic variation. Nature 2015;526:68–74. 26432245 10.1038/nature15393 PMC4750478

[22] NHLBI GO Exome Sequencing Project (ESP) . Exome Variant Server. Accessed November 10, 2018. Available at: https://evs.gs.washington.edu/EVS/.

[23] Lek M , Karczewski KJ , Minikel EV , et al. Analysis of protein‐coding genetic variation in 60,706 humans. Nature 2016;536:285–291. 27535533 10.1038/nature19057 PMC5018207

[24] Beaulieu CL , Majewski J , Schwartzentruber J , et al. FORGE Canada Consortium: outcomes of a 2‐year national rare‐disease gene‐discovery project. Am J Hum Genet 2014;94:809–817. 24906018 10.1016/j.ajhg.2014.05.003 PMC4121481

[25] GTEx Consortium . Human genomics. The Genotype‐Tissue Expression (GTEx) pilot analysis: multitissue gene regulation in humans. Science 2015;348:648–660. 25954001 10.1126/science.1262110 PMC4547484

[26] Johnson WE , Li C , Rabinovic A . Adjusting batch effects in microarray expression data using empirical Bayes methods. Biostatistics 2007;8:118–127. 16632515 10.1093/biostatistics/kxj037

[27] Langfelder P , Horvath S . WGCNA: an R package for weighted correlation network analysis. BMC Bioinformatics 2008;9:559. 19114008 10.1186/1471-2105-9-559 PMC2631488

[28] Botía JA , Vandrovcova J , Forabosco P , et al. An additional k‐means clustering step improves the biological features of WGCNA gene co‐expression networks. BMC Syst Biol 2017;11:47. 28403906 10.1186/s12918-017-0420-6 PMC5389000

[29] Reimand J , Kull M , Peterson H , et al. g:Profiler—a Web‐based toolset for functional profiling of gene lists from large‐scale experiments. Nucleic Acids Res 2007;35(Web Server issue):W193–W200. 17478515 10.1093/nar/gkm226 PMC1933153

[30] Shannon P , Markiel A , Ozier O , et al. Cytoscape: a software environment for integrated models of biomolecular interaction networks. Genome Res 2003;13:2498–2504. 14597658 10.1101/gr.1239303 PMC403769

[31] Malik K. Chemometric and quantum mechanical methods for analysing CD spectra. London, UK: University of London, 1997.

[32] Gorry PA . General least‐squares smoothing and differentiation by the convolution (Savitzky‐Golay) method. Anal Chem 1990;62:570–573.

[33] Hands‐Taylor KL , Martino L , Tata R , et al. Heterodimerization of the human RNase P/MRP subunits Rpp20 and Rpp25 is a prerequisite for interaction with the P3 arm of RNase MRP RNA. Nucleic Acids Res 2010;38:4052–4066. 20215441 10.1093/nar/gkq141 PMC2896528

[34] Romero‐Calvo I , Ocón B , Martínez‐Moya P , et al. Reversible Ponceau staining as a loading control alternative to actin in Western blots. Anal Biochem 2010;401:318–320. 20206115 10.1016/j.ab.2010.02.036

[35] Wilson MP , Footitt EJ , Papandreou A , et al. An LC‐MS/MS‐based method for the quantification of pyridox(am)ine 5'‐phosphate oxidase activity in dried blood spots from patients with epilepsy. Anal Chem 2017;89:8892–8900. 28782931 10.1021/acs.analchem.7b01358 PMC5588098

[36] Scott TA , Quintaneiro LM , Norvaisas P , et al. Host‐microbe co‐metabolism dictates cancer drug efficacy in *C. elegans* . Cell 2017;169:442–456.e18. 28431245 10.1016/j.cell.2017.03.040 PMC5406385

[37] Chalmers RM , Riordan‐Eva P , Wood NW . Autosomal recessive inheritance of hereditary motor and sensory neuropathy with optic atrophy. J Neurol Neurosurg Psychiatry 1997;62:385–387. 9120454 10.1136/jnnp.62.4.385 PMC1074097

[38] Zeisel A , Hochgerner H , Lönnerberg P , et al. Molecular architecture of the mouse nervous system. Cell 2018;174:999–1014.e22. 30096314 10.1016/j.cell.2018.06.021 PMC6086934

[39] Musayev FN , di Salvo ML , Ko TP , et al. Crystal structure of human pyridoxal kinase: structural basis of M(+) and M(2+) activation. Protein Sci 2007;16:2184–2194. 17766369 10.1110/ps.073022107 PMC2204131

[40] Gandhi AK , Desai JV , Ghatge MS , et al. Crystal structures of human pyridoxal kinase in complex with the neurotoxins, ginkgotoxin and theophylline: insights into pyridoxal kinase inhibition. PLoS One 2012;7:e40954. 22879864 10.1371/journal.pone.0040954 PMC3412620

[41] Kelly SM , Jess TJ , Price NC . How to study proteins by circular dichroism. Biochim Biophys Acta 2005;1751:119–139. 16027053 10.1016/j.bbapap.2005.06.005

[42] Flanagan JM , Beutler E . The genetic basis of human erythrocyte pyridoxal kinase activity variation. Haematologica 2006;91:801–804. 16704963

[43] Gisslén M , Price RW , Andreasson U , et al. Plasma concentration of the neurofilament light protein (NFL) is a biomarker of CNS injury in HIV infection: a cross‐sectional study. EBioMedicine 2015;3:135–140. 26870824 10.1016/j.ebiom.2015.11.036 PMC4739412

[44] Sandelius Å , Zetterberg H , Blennow K , et al. Plasma neurofilament light chain concentration in the inherited peripheral neuropathies. Neurology 2018;90:e518–e524. 29321234 10.1212/WNL.0000000000004932 PMC5818017

[45] Vrolijk MF , Opperhuizen A , Jansen EHJM , et al. The vitamin B6 paradox: supplementation with high concentrations of pyridoxine leads to decreased vitamin B6 function. Toxicol In Vitro 2017;44:206–212. 28716455 10.1016/j.tiv.2017.07.009

[46] Footitt EJ , Clayton PT , Mills K , et al. Measurement of plasma B6 vitamer profiles in children with inborn errors of vitamin B6 metabolism using an LC‐MS/MS method. J Inherit Metab Dis 2013;36:139–145. 22576361 10.1007/s10545-012-9493-y

[47] Darin N , Reid E , Prunetti L , et al. Mutations in PROSC Disrupt Cellular Pyridoxal Phosphate Homeostasis and Cause Vitamin‐B6‐Dependent Epilepsy. Am J Hum Genet 2016;99:1325–1337. 27912044 10.1016/j.ajhg.2016.10.011 PMC5142116

[48] Mills PB , Surtees RA , Champion MP , et al. Neonatal epileptic encephalopathy caused by mutations in the PNPO gene encoding pyridox(am)ine 5'‐phosphate oxidase. Hum Mol Genet 2005;14:1077–1086. 15772097 10.1093/hmg/ddi120

[49] Mills PB , Struys E , Jakobs C , et al. Mutations in antiquitin in individuals with pyridoxine‐dependent seizures. Nat Med 2006;12:307–309. 16491085 10.1038/nm1366

[50] Pena IA , Roussel Y , Daniel K , et al. Pyridoxine‐dependent epilepsy in zebrafish caused by Aldh7a1 deficiency. Genetics 2017;207:1501–1518. 29061647 10.1534/genetics.117.300137 PMC5714462

[51] Darin N , Reid E , Prunetti L , et al. Mutations in PROSC disrupt cellular pyridoxal phosphate homeostasis and cause vitamin‐B6‐dependent epilepsy. Am J Hum Genet 2016;99:1325–1337. 27912044 10.1016/j.ajhg.2016.10.011 PMC5142116

[52] Johnstone DL , Al‐Shekaili HH , Tarailo‐Graovac M , et al. PLPHP deficiency: clinical, genetic, biochemical, and mechanistic insights. Brain 2019;142:542–559. 30668673 10.1093/brain/awy346 PMC6391652

[53] Meisler NT , Thanassi JW . Pyridoxine kinase, pyridoxine phosphate phosphatase and pyridoxine phosphate oxidase activities in control and B‐6‐deficient rat liver and brain. J Nutr 1980;110:1965–1975. 6252301 10.1093/jn/110.10.1965

[54] Lumeng L , Ryan MP , Li TK . Validation of the diagnostic value of plasma pyridoxal 5'‐phosphate measurements in vitamin B6 nutrition of the rat. J Nutr 1978;108:545–553. 632943 10.1093/jn/108.4.545

[55] Whittaker JW . Intracellular trafficking of the pyridoxal cofactor. Implications for health and metabolic disease. Arch Biochem Biophys 2016;592:20–26. 26619753 10.1016/j.abb.2015.11.031 PMC4753123

[56] Wong HH , Lin JQ , Ströhl F , et al. RNA docking and local translation regulate site‐specific axon remodeling in vivo. Neuron 2017;95:852–868.e8 28781168 10.1016/j.neuron.2017.07.016 PMC5563073

[57] Cioni JM , Koppers M , Holt CE . Molecular control of local translation in axon development and maintenance. Curr Opin Neurobiol 2018;51:86–94. 29549711 10.1016/j.conb.2018.02.025

[58] Shigeoka T , Jung H , Jung J , et al. Dynamic axonal translation in developing and mature visual circuits. Cell 2016;166:181–192. 27321671 10.1016/j.cell.2016.05.029 PMC4930487

[59] Xu WY , Gu MM , Sun LH . A nonsense mutation in DHTKD1 causes Charcot‐Marie‐Tooth disease type 2 in a large Chinese pedigree. Am J Hum Genet 2012;91:1088–1094. 23141294 10.1016/j.ajhg.2012.09.018 PMC3516600

[60] Xu W , Zhu H , Gu M , et al. DHTKD1 is essential for mitochondrial biogenesis and function maintenance. FEBS Lett 2013;587:3587–3592. 24076469 10.1016/j.febslet.2013.08.047

[61] Gilman S. Joint position sense and vibration sense: anatomical organisation and assessment. J Neurol Neurosurg Psychiatry 2002;73:473–477. 12397137 10.1136/jnnp.73.5.473 PMC1738112

[62] di Salvo ML , Contestabile R , Safo MK . Vitamin B(6) salvage enzymes: mechanism, structure and regulation. Biochim Biophys Acta 2011;1814:1597–1608. 21182989 10.1016/j.bbapap.2010.12.006

[63] di Salvo ML , Safo MK , Contestabile R . Biomedical aspects of pyridoxal 5'‐phosphate availability. Front Biosci (Elite Ed) 2012;4:897–913. 22201923 10.2741/E428

[64] Clayton PT . B6‐responsive disorders: a model of vitamin dependency. J Inherit Metab Dis 2006;29:317–326. 16763894 10.1007/s10545-005-0243-2

[65] Gandhi AK , Ghatge MS , Musayev FN , et al. Kinetic and structural studies of the role of the active site residue Asp235 of human pyridoxal kinase. Biochem Biophys Res Commun 2009;381:12–15. 19351586 10.1016/j.bbrc.2009.01.170

[66] Albersen M , Bosma M , Luykx JJ , et al. Vitamin B‐6 vitamers in human plasma and cerebrospinal fluid. Am J Clin Nutr 2014;100:587–592. 24808484 10.3945/ajcn.113.082008

