# Supplementary material for: PDXK mutations cause polyneuropathy responsive to pyridoxal 5′‐phosphate supplementation
Source: Ann Neurol. 2019 Jul 1;86(2):225–40. doi: 10.1002/ana.25524 (PMC6772106; doi:10.1002/ana.25524)
Supplement: Supplementary file 1 — Supplementary Table 1 Extended neurological examination pre and on PLP supplementation in patients with PDXK mutations. Supplementary Table 2. Electrophysiological studies in patients with PDXK mutations. Supplementary Table 3. Gene co‐expression network analysis for PDXK. Supplementary Table 4. Description of the genotype of promoter region for the control group and PDXK p.Ala228Thr cases. Supplementary Table 5. Clinical response to pyridoxal 5’‐phosphate (PLP) treatment. Supplementary Table 6. SYNaPS Study Group collaborators. [file ANA-86-225-s001.docx]

**Supplementary Table 1**. Extended neurological examination pre and on PLP supplementation in patients with *PDXK* mutations.

| **Case/ Time of evaluation** | **F1-II-5 baseline** | | **F1-II-5 on PLP** | | **F1-II-6 baseline** | | **F1-II-6 on PLP** | | **F2-II-1 baseline** | | **F2-II-2 baseline** | |
| --- | --- | --- | --- | --- | --- | --- | --- | --- | --- | --- | --- | --- |
| Face | Normal examination | | Normal examination | | Normal examination | | Normal examination | | Normal examination | | Normal examination | |
| Neck Flexion |  |  |  |  |  |  |  |  |  |  |  |  |
| Neck Extension |  |  |  |  |  |  |  |  |  |  |  |  |
| **Muscle assessed/ Side** | R | L | R | L | R | L | R | L | R | L | R | L |
| Shoulder Abduction | 5 | 5 | 5 | 5 | 5 | 5 | 5 | 5 | 5 | 5 | 5 | 5 |
| (Supraspinatus) | 5 | 5 | 5 | 5 | 5 | 5 | 5 | 5 | 5 | 5 | 5 | 5 |
| Deltoid | 5 | 5 | 5 | 5 | 5 | 5 | 5 | 5 | 5 | 5 | 5 | 5 |
| Shoulder Adduction | 5 | 5 | 5 | 5 | 5 | 5 | 5 | 5 | 5 | 5 | 5 | 5 |
| Shoulder External Rotation | 5 | 5 | 5 | 5 | 5 | 5 | 5 | 5 | 5 | 5 | 5 | 5 |
| Elbow Flexion | 4+ | 4+ | **5** | **5** | 5 | 5 | 5 | 5 | 5 | 5 | 5 | 5 |
| Elbow Extension | 4 | 4 | **5** | **5** | 5 | 5 | 5 | 5 | 5 | 5 | 5 | 5 |
| Wrist Extension | 4 | 4 | **5** | **5** | 5 | 5 | 5 | 5 | 5 | 5 | 5 | 5 |
| Wrist Flexion | 4 | 4 | **5** | **5** | 4+ | 4+ | 4+ | 4+ | 5 | 5 | 5- | 5- |
| Extensor Dig Comm | 4 | 4 | **5** | **5** | 4- | 4- | 4- | 4- | 4 | 4 | 4+ | 4+ |
| Flexor digitorum superficialis | 4 | 4 | **4+** | **4+** | na | na | na | na | 5 | 5 | 5 | 5 |
| Flexor digitorum profundus | 4 | 4 | **4+** | **4+** | na | na | na | na | 5 | 5 | 5 | 5 |
| First dorsal interosseous | 3 | 3 | 3 | 3 | 3 | 3 | 3 | 3 | 4+ | 4+ | 4 | 4 |
| Abductor pollicis brevis | 3 | 3 | 3 | 3 | 3 | 3 | 3 | 3 | 4 | 4 | 4 | 4 |
| Abductor digiti minimi | 3 | 3 | 3 | 3 | 3 | 3 | 3 | 3 | 4+ | 4 | 4- | 4 |
| Hip Flexion | 4+ | 4+ | **5** | **5** | 5 | 5 | 5 | 5 | 5 | 5 | 5 | 5 |
| Hip Extension | 4+ | 4+ | **5** | **5** | 5 | 5 | 5 | 5 | 5 | 5 | 5 | 5 |
| Knee Flexion | 4- | 4- | **5** | **5** | 5 | 5 | 5 | 5 | 5 | 5 | 5 | 5 |
| Knee Extension | 4- | 4- | **5** | **5** | 5 | 5 | 5 | 5 | 5 | 5 | 5 | 5 |
| Ankle Dorsiflexion | 1 | 1 | **1** | **1** | 0 | 0 | 0 | 0 | 3 | 3 | 2 | 2 |
| Ankle Plantarflexion | 0 | 0 | 0 | 0 | 0 | 0 | 0 | 0 | 5 | 5 | 5 | 5 |
| Toe Flexion | 0 | 0 | 0 | 0 | 0 | 0 | 0 | 0 | 1 | 1 | 4 | 4- |
| **MRC sum Score (60)** | **44** | | **52** | | **44** | | **50** | | **56** | | **54** | |
| **Reflexes** | | | | | | | | | | | | |
| Biceps | Abs | Abs | Abs | Abs | Abs | Abs | Abs | Abs | Abs | Abs | Abs | Abs |
| Supinator | Abs | Abs | Abs | Abs | Abs | Abs | Abs | Abs | Abs | Abs | Abs | Abs |
| Triceps | Abs | Abs | Abs | Abs | Abs | Abs | Abs | Abs | Abs | Abs | Abs | Abs |
| Knee | Abs | Abs | Abs | Abs | Abs | Abs | Abs | Abs | Abs | Abs | Abs | Abs |
| Ankle | Abs | Abs | Abs | Abs | Abs | Abs | Abs | Abs | Abs | Abs | Abs | Abs |
| Plantars | mute | mute | mute | mute | mute | mute | mute | mute | mute | mute | mute | mute |
| **Sensation** | | | | | | | | |  |  |  |  |
| Pinprick | UL: above wrists  LL: mid calf | UL: above wrists  LL: at ankles | UL: mid hand  LL: mid foot | UL: mid hand  LL: mid foot | UL: At elbows  LL: ankles | UL: At elbows  LL: at ankles | UL: At elbows  LL: at ankles | UL: At elbows  LL: at ankles | UL: MCP  LL: lower calf | UL: MCP  LL: lower calf | UL: MCP LL: lower calf | UL: MCP  LL: lower calf |
| Vibration | UL: At wrists  LL: iliac crest | UL: At wrists  LL: iliac crest | UL: At wrists  LL: iliac crest | UL: At wrists  LL: iliac crest | UL: At elbows  LL: iliac crest | UL: At elbows  LL: iliac crest | UL: At elbows  LL: iliac crest | UL: At elbows  LL: iliac crest | UL: N  LL: MM | UL: N  LL: MM | UL: N  LL: MM | UL: N  LL: r patella |

Legend: abs=absent, R=right, L=left, UL=upper limb, LL=lower limb, na=not available, MCP= metacarpophalangeal joints, MM=medial malleolus. The movements assessed as part of the MRC sum power score are highlighted in grey. The values that have improved on PLP treatment are highlighted in green.

**Supplementary Table 2.** Electrophysiological studies in patients with *PDXK* mutations

| **Time of test** | **Motor nerve conduction studies** | | | | | | | | | | | | | | | | | | | | | | | | | | | | | | | | | | | |
| --- | --- | --- | --- | --- | --- | --- | --- | --- | --- | --- | --- | --- | --- | --- | --- | --- | --- | --- | --- | --- | --- | --- | --- | --- | --- | --- | --- | --- | --- | --- | --- | --- | --- | --- | --- | --- |
|  | **Median nerve** | | | | | | | | | | | | **Ulnar nerve** | | | | | | | | | | | | | | | | **Common Peronial nerve** | | | | **Tibial nerve** | | | |
|  | **DML** | **CMAP (w.)** | | **CMAP (e.)** | | **MCV**  **(w. to e.)** | | **MCV**  **(e. to a)** | | **F lat** | | | **DML** | | **CMAP (w.)** | | | **CMAP (e.)** | | **CMAP (e. to a)** | | **MCV w. to e.** | | **MCV**  **e. to a.** | | **F lat** | | | **CMAP (ank)** | | **CMAP (fib. neck)** | | **CMAP pop.fossa)** | | | **CMAP (ank)** |
| **F1-II-5** | | | | | | | | | | | | | | | | | | | | | | | | | | | | | | | | | | | | |
| **-20 years** | 4.5 | 4.7 | | 5.0 | | 44 | | 64 | | 33.4 | | | 3.3 | | 5.0 | | | 4.3 | | 3.7 | | 44 | | 56 | | | 34.6 | | NR | | NR | | NR | | | NR |
| **-7 years** | 5.3 | 0.6 | | 0.5 | | 35 | | na | | 32.5 | | | 3.3 | | 2.2 | | | 1.5 | | 1.5 | | 43 | | 48 | | | 35.9 | | NR | | NR | | NR | | | NR |
| **Baseline** | 6.1 | 0.4 | | 0.3 | | 35 | | na | | abs | | | 3.6 | | 2.1 | | | 2.0 | | 2.0 | | 44 | | 51 | | | 36 | | NR | | NR | | NR | | | NR |
| **After PLP** | 6.2 | 0.3 | | 0.2 | | 28 | | na | | abs | | | 3.1 | | 2.6 | | | 2.0 | | 1.7 | | 36.4 | | 42 | | | 41 | | NR | | NR | | NR | | | NR |
| **F1-II-6** | | | | | | | | | | | | | | | | | | | | | | | | | | | | | | | | | | | | |
| **Baseline** | 5.9 | 0.1 | | 0.1 | | 38 | | na | | na | | | 3.8 | | 1.8 | | | 1.5 | | 1.5 | | 41 | | 45 | | | 37 | | NR | | NR | | na | | NR | |
| **After PLP** | 5.8 | 0.32 | | 0.25 | | 32.4 | | na | | na | | | 3.21 | | 2.3 | | | 1.74 | | 1.78 | | 42.3 | | 50 | | | na | | NR | | NR | | NR | | NR | |
| **F2-II-1** | | | | | | | | | | | | | | | | | | | | | | | | | | | | | | | | | | | | |
| Baseline | 3.23 | 7.1 | | 5.3 | | 39.9 | | na | | na | | | 2.92 | | 6.0 | | | 5.7 | | 5.8 | | 56.3 | | 54.9 | | | na | | NR | | NR | | NR | | NR | |
| **F2-II-2** | | | | | | | | | | | | | | | | | | | | | | | | | | | | | | | | | | | | |
| Baseline | 3.96 | 11.8 | | 9.4 | | 46.2 | | na | | na | | | 3.39 | | 11.1 | | | 8.2 | | 8.3 | | 44.5 | | 49.2 | | | na | | NR | | NR | | NR | | NR | |
|  | | | | | | | | | | | | | | | | | | | | | | | | | | | | | | | | | | | | |
| **Time of test** | **Sensory and mixed nerve conduction studies** | | | | | | | | | | | | | | | **Visual evoked potentials** | | | | | | | | | | | | | | | | | | | | |
|  | **Radial nerve** | | **Median nerve** | | | | **Ulnar nerve** | | | | | **Sural Nerve** | | | | **Eye** | | | | | **Right eye** | | | | **Left eye** | | | | | **Right eye** | | | **Left eye** | | | |
|  |  |  |  |  |  |  |  |  |  |  |  |  |  |  |  | **Time of test** | | | **P100** | | **uV** | | **msec** | | **uV** | | | **msec** | | **uV** | | **msec** | **uV** | **msec** | | |
|  | **SNAP (uV)** | **SCV (m/s)** | **SNAP (uV)** | | **SCV (m/s)** | | **SNAP (uV)** | | **SCV (m/s)** | | | **SNAP (uV)** | | **SCV (m/s)** | |  |  |  | **F1-II-5** | | | | | | | | | | | **F1-II-6** | | | | | | |
| **F1-II-5** | | | | | | | | | | | | | | | | **-20 years** | | | **Visual acuity** | | 6/9 | | | | 6/24 | | | | | 6/12 | | | 6/9+1 | | | |
| -20 years | NR | **-** | NR | | **-** | | NR | | **-** | | NR | | | **-** | |  |  |  | Whole f. | | 2 | | 106 | | 1.7 | | | 108 | | **7.9** | | 101 | **7.4** | 100 | | |
| Baseline | NR | **-** | NR | | **-** | | NR | | **-** | | NR | | | **-** | |  |  |  |  |  |  |  |  |  |  |  |  |  |  |  |  |  |  |  |  |  |
| After PLP | NR | **-** | NR | | **-** | | NR | | **-** | | NR | | | **-** | |  |  |  | R. hemif. | | na | | na | | na | | | na | | 2.8 | | 97.1 | 3.3 | 91.6 | | |
| **F1-II-6** | | | | | | | | | | | | | | | |  |  |  | L. hemif. | | na | | na | | na | | | na | | 2.7 | | 94.6 | 2.3 | 100 | | |
| Baseline | NR | **-** | NR | | **-** | | NR | | **-** | | NR | | | **-** | |  |  |  | Central f. | | na | | na | | na | | | na | | 6.7 | | 101 | 6.5 | 100 | | |
| After PLP | NR | **-** | NR | | **-** | | NR | | **-** | | NR | | | **-** | | **Baseline** | | | **Visual acuity** | | NA | | | | | | | | | 6/12 | | | 6/18 | | | |
| **F2-II-1** | | | | | | | | | | | | | | | |  |  |  | Whole f. | |  |  |  |  |  |  |  |  |  | 1.9 | | 109 | 2 | 110 | | |
| Baseline | NR | - | NR | | **-** | | NR | | **-** | | NR | | | **-** | |  |  |  | Right hemif. | |  |  |  |  |  |  |  |  |  | na | | na | na | na | | |
| **F2-II-2** | | | | | | | | | | | | | | | |  |  |  | L. hemif. | |  |  |  |  |  |  |  |  |  | na | | na | na | na | | |
| Baseline | NR | - | NR | | **-** | | NR | | **-** | | NR | | | **-** | |  |  |  | Central f. | |  |  |  |  |  |  |  |  |  | na | | na | na | na | | |
|  | | | | | | | | | | | | | | | | | | | | | | | | | | | | | | | | | | | | |
| **Somatosensory evoked potentials** | | | | | | | | | | | | | | | | |  | | | | | | | | | | | | | | | | | | | |

| **Site** | | **Right** | | | | | **Left** | | | |  | | | | | | | | | |
| --- | --- | --- | --- | --- | --- | --- | --- | --- | --- | --- | --- | --- | --- | --- | --- | --- | --- | --- | --- | --- |
| Tibial (twitch) | | uV | | ms | | | uV | | ms | |  |  |  |  |  |  |  |  |  |  |
| P40 (scalp) | | 0.2 | | 45.3 | | |  | | indistinct | |  |  |  |  |  |  |  |  |  |  |
| N22 (T12/L1) | |  | | indistinct | | |  |  | indistinct | |  |  |  |  |  |  |  |  |  |  |
| N8 (popliteal fossa) | |  |  | indistinct | | |  |  | indistinct | |  |  |  |  |  |  |  |  |  |  |
| IPL N8-P22 | | na | |  | | | na | |  | |  |  |  |  |  |  |  |  |  |  |
| IPL N22-P40 | | na | |  |  |  | na | |  |  |  |  |  |  |  |  |  |  |  |  |
|  | | | | | | | | | | | | | | | | | | | | |
| **Electromyography** | | | | | | | | | | | | | | | | | | | | |
| **Site** | **Spontaneous activity** | | | | **MUAP configuration** | | | | | **Recruit.** | | **Interfer.** | **Spontaneous activity** | | **MUAP configuration** | | | | **Recruit.** | **Interfer.** |
|  | **Fibs/**  **PSW** | | **Other** | | **Dur** | **Amp** | | **Poly** | |  |  |  | **Fibs/PSW** | **Other** | **Dur** | **Amp** | **Poly** | |  |  |
| **F1-II-5 baseline** | | | | | | | | | | | | | **F1-II-5 Post PLP** | | | | | | | |
| **Right Biceps** | 0 | | 0 | | 🡹 | 🡹 | | 🡹 | | N | | N | 0 | 0 | 🡹 | 🡹 | 🡹 | | N | N |
| **Flexor carpi radialis** | 0 | | 0 | | 🡹 | 🡹 | | 🡹 | | N | | N | 0 | 0 | 🡹 | 🡹 | 🡹 | | N | N |
| **Flexor Digitalis superficialis** | 0 | | 0 | | 🡹🡹 | 🡹🡹 | | 🡹🡹 | | Reduced recruitment | | 🡻 | 0 | 0 | 🡹🡹 | 🡹🡹 | 🡹🡹 | | Reduced recruitment | 🡻 |
| **Right FDIO** | 0 | | 0 | | 🡹🡹 | 🡹🡹 | | 🡹🡹 | | Reduced recruitment | | 🡻🡻🡻 | 0 | 0 | 🡹🡹 | 🡹🡹 | 🡹🡹 | | Reduced recruitment | 🡻🡻🡻 |
| **Right Vast**  **lateralis** | 0 | | 0 | | 🡹🡹 | 🡹🡹 | | 🡹🡹 | | Reduced recruitment | | 🡻🡻 | 0 | 0 | 🡹 | 🡹 | 🡹 | | Reduced recruitment | 🡻 |
| **Right Tib Ant** | 0 | | 0 | | na | na | | na | | No MUAPs under voluntary control | | na | 0 | 0 | 🡹🡹 | 🡹🡹 | 🡹 | | Reduced recruitment. | 🡻🡻 |
| **F1-II-6 baseline** | | | | | | | | | | | | | **F1-II-6 Post PLP** | | | | | | | |
| **Right Biceps** | 0 | | 0 | | N | N | | N | | N | | N | 0 | 0 | N | N | N | N | | N |
| **Flexor carpi radialis** | 0 | | 0 | | 🡹 | 🡹 | | 🡹 | | N | | N | 0 | 0 | 🡹 | 🡹 | 🡹 | N | | N |
| **Flexor Digitalis superficialis** | 0 | | 0 | | 🡹 | 🡹 | | 🡹 | | N | | 🡻 | 0 | 0 | 🡹 | 🡹 | 🡹 | N | | 🡻 |
| **Right FDIO** | 0 | | 0 | | 🡹 | 🡹 | | 🡹 | | N | | 🡻🡻🡻 | 0 | 0 | 🡹 | 🡹 | 🡹 | N | | 🡻🡻🡻 |
| **Right Vast**  **lateralis** | 0 | | 0 | | 🡹🡹 | 🡹🡹 | | 🡹🡹 | | Reduced recruitment | | 🡻🡻 | 0 | 0 | 🡹🡹 | 🡹🡹 | 🡹🡹 | Reduced recruitment | | 🡻 |
| **Right Tib Ant** | 0 | | 0 | | N | N | | N | | No MUAPs under voluntary control | | na | 0 | 0 | N | N | N | Reduced recruitment. | | 🡻🡻 |
| **F2-II-1 baseline** | | | | | | | | | | | | |  | | | | | | | |
| **Right FDIO** | 0 | | 0 | | N | 🡹 | | N | | Mild reduced recruitment | | N |  |  |  |  |  |  |  |  |
| **Right Tib Ant** | 0 | | 0 | | 🡹🡹 | 🡹🡹 | | N | | Reduced recruitment | | 🡻🡻🡻 |  |  |  |  |  |  |  |  |

Electrophysiological studies in patients with *PDXK* mutations. Motor and sensory nerve conduction studies (NCS) in patients with *PDXK* mutation show a sensorimotor, axonal neuropathy progressing in a length-dependent pattern. Studies were carried out 20 and 7 years before starting PLP supplementation, at baseline (just before PLP supplementation) and 1 year post PLP replacement in case F1-II-5 and F1-II-6 and at baseline in F2-II-1. Longitudinal assessment of visual evoked potentials (VEP) in patients with *PDXK* mutations before PLP treatment demonstrate that responses to pattern reversal stimulation (case F1-II-5) to either eye were of very low amplitude. Whole field responses were just discernable, with normal latencies, but responses to stimulation of the constituent parts of the visual field could not be identified. Although initially normal, longitudinal assessment of the second affected patient (F1-II-6) 20 years into the disease showed similarly abnormal VEP of both eyes and were severely attenuated but undelayed consistent with axonal loss and bilateral visual pathway involvement. Stimulation was performed with a CRT monitor using a black and white square pattern with a contrast of 97%, a mean luminance of 61 cd/m^2^ (photopic candelas seconds per meter squared) and a reversal rate of 2.1/s. Recordings of the evoked responses used electrodes at MO (midoccipital), LO (lateral occipital) and RO (right occipital) (recording), Fz (reference) and Cz (ground) and filters at 1Hz (HP) and 100 Hz (LP). Amplitudes were measured from N75 to P100 (peak latency of the waveform) and latencies to the peak of P100 in recordings from MO. Somatosensory evoked potentials (SEP) in case F1-II-6 at baseline from the right lower limb shows very small and poorly formed cortical responses of normal latency. No responses were elicited from the left foot. These findings likely reflect the severe peripheral neuropathy rather than a central lesion. Unilateral electrical stimulation of the nerve was performed at 3.1/s. Latencies

were measured to peaks and amplitudes from peaks to following throughs or to P50 for cortical responses in lower limb studies. Electromyography (EMG) study in patients with *PDXK* mutations at baseline and after PLP supplementation with concentric needle EMG at baseline (before PLP supplementation) shows chronic denervation in a length-dependent pattern with no evidence of myopathy.

Legend: DML = distal motor latency measured in milliseconds; CMAP =Compound Muscle Action Potential, measured in milivolts; MCV= motor conduction velocity, measured in metres per second; F lat= Minimal F wave latency, measured in milliseconds; Term. Dist =terminal distances measured in millimetres; w=wrist, e.=elbow, a.=axilla, ank=ankle, NR = no response, na=not available, SNAP= sensory nerve action potential, measured in microvolts; SCV=sensory conduction velocity measured in metres per second, uV=microvolts, msec=milliseconds, f=field. Spontaneous activity: 0, none; 1, at two sites; 2, at more than 2 sites; 3, at all sites, 4, "interference pattern". MUAP configuration, Interference: N, normal; 🡹/🡻, mild, 🡹🡹 /🡻🡻, moderate, 🡹🡹🡹 /🡻🡻🡻, severe increase or decrease. Fibs= fibrillation potentials, PSW= positive sharp waves; MUAP= motor unit action potential; Dur=duration; Amp=amplitude; Poly=polypasic units; Recruit=Recruitment; Interfer=Interference; FDIO=first dorsal interosseos, Vast Med = vastus medialis, Tib Ant=tibial anterior muscles; na= not available.

**Supplementary Table 3. Gene co-expression network analysis for *PDXK*.**

Table shows the most enriched GO terms within each of the PDXK-containing modules generated using co-expression network analysis of GTEx central nervous system and peripheral nervous system transcriptomic data.

**Supplementary Table 4. Description of the genotype of promoter region for the control group and *PDXK* p.Ala228Thr cases.**

| ID | Age | Gender | Ethnicity | Genotype of promoter region  (Ins = insertion present;  WT = insertion absent) | PDXK activity in DBS (pmol DBS^-1^ hr^-1^) |
| --- | --- | --- | --- | --- | --- |
| 1 | 25 | Female | African | Ins ; Ins | 4.4 |
| 2 | 50 | Female | Caucasian | Ins ; Ins | 9.1 |
| 3 | 71 | Female | Indian | Ins ; WT | 4.2 |
| 4 | 50 | Female | Caucasian | Ins ; Ins | 4.2 |
| 5 | 49 | Female | Caucasian | Ins ; Ins | 7.8 |
| 6 | 48 | Male | Caucasian | Ins ; Ins | 8.7 |
| 7 | 30 | Male | Caucasian | WT ; WT | 6.0 |
| 8 | 70 | Female | Caucasian | Ins ; Ins | 9.0 |
| 9 | 35 | Female | East Asian | Ins ; Ins | 5.4 |
| 10 | 27 | Male | Caucasian | Ins ; Ins | 12.3 |
| 11 | 52 | Female | African | Ins ; WT | 2.6 |
| 12 | 71 | Female | Caucasian | WT ; WT | 10.1 |
| 13 | 92 | Male | Caucasian | Ins ; Ins | 9.8 |
| 14 | 55 | Male | South Asian | Ins ; Ins | 7.7 |
| 15 | 15 | Male | Caucasian | Ins ; Ins | 7.7 |
| 16 | 64 | Female | Caucasian | WT ; WT | 7.6 |
| 17 | 56 | Male | Caucasian | WT ; WT | 5.9 |
| 18 | 38 | Female | Caucasian | Ins ; Ins | 11.7 |
| 19 | 31 | Male | Arabic | Ins ; Ins | 14.7 |
| 20 | 34 | Male | Caucasian | Ins ; Ins | 10.1 |
| 21 | 78 | Female | Cypriot | Ins ; Ins | 9.0 |
| *PDXK* p.Ala228Thr homozygous cases | | | | | |
| 1 | 79 | Male | Cypriot | WT ; WT | 1.1 |
| 2 | 74 | Female | Cypriot | WT ; WT | 0.8 |
| *PDXK* p.Ala228Thr/WT case | | | | | |
| 1 | 52 | Female | Cypriot | Ins ; WT | 4.9 |
| *PDXK* p.Arg220Gln homozygous cases | | | | | |
| 1 | 31 | Female | Canadian | WT ; WT | 1.19 |
| 2 | 29 | Female | Canadian | WT ; WT | 0.5 |

**Supplementary Table 5.** Clinical response to pyridoxal 5’-phosphate (PLP) treatment.

| **Neurological impairment set** | | **Baseline, pre PLP** | | **1 year post PLP** | | **Baseline, pre PLP** | | **1 year post PLP** | |
| --- | --- | --- | --- | --- | --- | --- | --- | --- | --- |
|  |  | Case F1-II-5 | | | | Case F1-II-6 | | | |
| Motor | Left upper limb | 2 | | 2 | | 2 | | 2 | |
|  | Right upper limb | 2 | | 2 | | 2 | | 2 | |
|  | Left lower limb | 3 | | 2 | | 3 | | 2 | |
|  | Right lower limb | 3 | | 2 | | 3 | | 2 | |
|  | Trunk | 0 | | 0 | | 0 | | 0 | |
| Tone | | 0 | | 0 | | 0 | | 0 | |
| Sensation | | 3 | | 3 | | 3 | | 3 | |
| Perceptual function | | 0 | | 0 | | 0 | | 0 | |
| Speech and language | | 0 | | 0 | | 0 | | 0 | |
| Cognitive function | | 0 | | 0 | | 0 | | 0 | |
| Behaviour | | 0 | | 0 | | 0 | | 0 | |
| Mood | | 0 | | 0 | | 0 | | 0 | |
| Seeing and Vision | | 3 | | 3 | | 2 | | 2 | |
| Hearing | | 0 | | 0 | | 0 | | 0 | |
| Pain | | 2 | | 0 | | 1 | | 0 | |
| Fatigue | | 1 | | 0 | | 1 | | 0 | |
| Other | | 0 | | 0 | | 0 | | 0 | |
| **Total** | | **19** | | **14** | | **17** | | **13** | |
| **Charcot-Marie-Tooth neuropathy score** | |  | | | | | | | |
| Sensory symptoms | | 3 | | 3 | | 3 | | 3 | |
| Motor symptoms (legs) | | 4 | | **3** | | 3 | | 3 | |
| Motor symptoms (arms) | | 3 | | **2** | | 2 | | 2 | |
| Pinprick sensibility | | 3 | | 3 | | 3 | | 3 | |
| Vibration | | 4 | | 4 | | 4 | | 4 | |
| Strength (legs) | | 3 | | 3 | | 3 | | 3 | |
| Strength (arms) | | 3 | | **2** | | 3 | | **2** | |
| Ulnar CMAP | | 3 | | 2 | | 3 | | **2** | |
| Radial SAP amplitude, antidromic testing | | 4 | | 4 | | 4 | | 4 | |
| **Total** | | **30** | | **26** | | **28** | | **26** | |
| **MRC sum score** | |  | | | | | | | |
| **Muscle assessed/ Side** | | Right | Left | Right | Left | Right | Left | Right | Left |
| Shoulder Abduction | | 5 | 5 | 5 | 5 | 5 | 5 | 5 | 5 |
| Elbow Flexion | | 4+ | 4+ | **5** | **5** | 5 | 5 | 5 | 5 |
| Wrist Extension | | 4 | 4 | **5** | **5** | 4 | 4 | **5** | **5** |
| Hip Flexion | | 4+ | 4+ | **5** | **5** | 4 | 4 | **5** | **5** |
| Knee Extension | | 4- | 4- | **5** | **5** | 4+ | 4+ | **5** | **5** |
| Ankle Dorsiflexion | | 1 | 1 | **1** | **1** | 0 | 0 | 0 | 0 |
| **MRC sum Score (60)** | | **44** | | **52** | | **44** | | **50** | |

Three rating scales were used to assess the response to PLP supplementation: the Medical Research Council (MRC) sum score assessing the motor and power, the Charcot-Marie-Tooth neuropathy that combines motor, sensory and electrophysiology results and the Neurological Impairment score that combines a wide range of neurological function to assess diseases severity. PLP=pyridoxal 5’-phosphate, CMAP= compound muscle action potential, SAP= sensory nerve action potential.

**Supplementary Table 6.** SYNaPS Study Group collaborators

| Prof Yamna Kriouile | Affiliation: Unit of Neuropediatrics, Children's Hospital of Rabat, University of Rabat, Rabat 6527, Morocco  Email: [d](mailto:barakatamina@hotmail.fr)[r.kriouile@gmail.com](mailto:r.kriouile@gmail.com) |
| --- | --- |
| Prof. Mohamed El Khorassani | Affiliation: Children's Hospital of Rabat, University of Rabat, Rabat 6527, Morocco  Email: [elkhorassani.mohamed@gmail.com](mailto:elkhorassani.mohamed@gmail.com) |
| Prof. Mhammed Aguennouz | Affiliation: Department of Clinical and Experimental Medicine, University of Messina, Messina 98123, Italy  Email: [aguenoz@unime.it](mailto:aguenoz@unime.it) |
| Prof Stanislav Groppa | Affiliation: Department of Neurology and Neurosurgery, Institute of Emergency Medicine, Chisinau, Republic of Moldova.  Email: [sgroppa@gmail.com](mailto:sgroppa@gmail.com) |
| Dr. Blagovesta Marinova Karashova | Affiliation: Department of Paediatrics, Medical University of Sofia, Sofia 1431, Bulgaria Email: [blagovestakarashova@gmail.com](mailto:blagovestakarashova@gmail.com) |
| Prof Lionel Van Maldergem | Affiliation: Centre of Human Genetics, University Hospital Liege, Liege 4000, Belgium  Email:  [lvanmaldergem@chu-besancon.fr](mailto:lvanmaldergem@chu-besancon.fr) |
| Dr. Wolfgang Nachbauer | Affiliation: Department of Neurology, Medical University Innsbruck, Anichstrasse 35, Innsbruck 6020, Austria  Email: Wolfgang.Nachbauer@i-med.ac.at |
| Prof. Sylvia Boesch | Affiliation: Department of Neurology, Medical University Innsbruck, Anichstrasse 35, Innsbruck 6020, Austria  Email: [sylvia.boesch@i-med.ac.at](mailto:sylvia.boesch@i-med.ac.at) |
| Dr. Larissa Arning | Affiliation: Department of Human Genetics, Ruhr-University Bochum, Bochum 44801, Germany  Email: [Larissa.Arning@ruhr-uni-bochum.de](mailto:Larissa.Arning@ruhr-uni-bochum.de) |
| Prof. Dagmar Timmann | Affiliation: Braun Neurologische Universitätsklinik Universität Essen, Hufelandstr 55, Essen D-45122, Germany  Email: [Dagmar.Timmann-Braun@uni-duisburg-essen.de](mailto:Dagmar.Timmann-Braun@uni-duisburg-essen.de) |
| Prof. Bru Cormand | Affiliation: Department of Genetics, Universitat de Barcelona, Barcelona 08007, Spain  Email: bcormand@ub.edu |
| Dr. Belen Pérez-Dueñas | Affiliation: Hospital Sant Joan de Deu, Esplugues de Llobregat 08950, Barcelona, Spain  Email: bperez@sjdhospitalbarcelona.org |
| Dr Gabriella Di Rosa, MD, PhD | Affiliation: Department of Pediatrics, University of Messina, Messina 98123, Italy  Email: gdirosa@unime.it |
| Prof. Jatinder S. Goraya, MD, FRCP | Affiliation: Division of Paediatric Neurology, Dayanand Medical College & Hospital, Ludhiana, Punjab 141001, India  Email: gorayajs@gmail.com |
| Prof. Tipu Sultan | Affiliation: Division of Paediatric Neurology, Children's Hospital of Lahore, Lahore 381-D/2, Pakistan  Email: [tipusultanmalik@hotmail.com](mailto:tipusultanmalik@hotmail.com) |
| Prof Jun Mine | Affiliation: Department of Paediatrics, Shimane University, Faculty of Medicine, Izumo, 693-8501, Japan  Email:  [jmine@med.shimane-u.ac.jp](mailto:jmine@med.shimane-u.ac.jp) |
| Prof. Daniela Avdjieva, | Affiliation: Department of Paediatrics, Medical University of Sofia, Sofia 1431, Bulgaria  Email: [davdjieva@yahoo.com](mailto:davdjieva@yahoo.com) |
| Dr. Hadil Kathom, | Affiliation: Department of Pediatrics, Medical University of Sofia, Sofia 1431, Bulgaria  Email: [hadilmk@gmail.com](mailto:hadilmk@gmail.com) |
| Prof.Dr Radka Tincheva | Affiliation: Head of Department of Clinical Genetics, University Pediatric Hospital, Sofia 1431, Bulgaria  Email: [radka.tincheva@gmail.com](mailto:radka.tincheva@gmail.com) |
| Prof. Selina Banu | Affiliation: Neurosciences Unit, Institute of Child Health and Shishu Shastho Foundation Hospital, Mirpur, Dhaka 1216, Bangladesh  Email: [selinabanu17@gmail.com](mailto:selinabanu17@gmail.com) |
| Prof. Mercedes Pineda-Marfa | Affiliation Servei de Neurologia Pediàtrica, l'Hospital Universitari Vall d'Hebron, Barcelona 08035, Spain  Email: [pineda@hsjdbcn.org](mailto:pineda@hsjdbcn.org) |
| Prof. Pierangelo Veggiotti | Affiliation: Unit of Infantile Neuropsychiatry Fondazione  Istituto Neurologico "C. Mondino" IRCCS, Via Mondino 2, Pavia 27100, Italy  Email: [pierangelo.veggiotti@unipv.it](mailto:pierangelo.veggiotti@unipv.it) |
| Prof. Michel D. Ferrari | Affiliation: Leiden University Medical Center, Albinusdreef 2, Leiden 2333, Netherlands  Email: M.D.Ferrari@lumc.nl |
| Prof [Arn M J M van den Maagdenberg](https://www.google.co.uk/url?sa=t&rct=j&q=&esrc=s&source=web&cd=4&cad=rja&uact=8&ved=0ahUKEwjBjPHRx_bSAhVMKsAKHeivBpEQFggtMAM&url=https%3A%2F%2Fwww.researchgate.net%2Fprofile%2FArn_Maagdenberg&usg=AFQjCNFXE-qX1wsjWLQQjl7yy5M-uMdLRQ) | Affiliation: Leiden University Medical Center, Albinusdreef 2, Leiden 2333, Netherlands  [A.M.J.M.van_den_Maagdenberg@lumc.nl](mailto:A.M.J.M.van_den_Maagdenberg@lumc.nl) |
| Prof. Alberto Verrotti | Affiliation: University of L’Aquila, L’Aquila, Italy  Email: [verrottidipianella@univaq.it](mailto:verrottidipianella@univaq.it) |
| Prof Giangluigi Marseglia | Affiliation: Department of Pediatrics, University of Pavia, IRCCS Policlinico "San Matteo", Pavia 27100, Italy  Email: [gl.marseglia@smatteo.pv.it](mailto:gl.marseglia@smatteo.pv.it) |
| Dr. Salvatore Savasta | Affiliation: Division of Pediatric Neurology, Department of Pediatrics, University of Pavia, IRCCS Policlinico "San Matteo", Pavia 27100, Italy  Email: [S.Savasta@smatteo.pv.it](mailto:S.Savasta@smatteo.pv.it) |
| Dr. Mayte García-Silva | Affiliation: Hospital Universitario 12 de Octubre, Madrid 28041, Spain  Email: [mgarciasilva@salud.madrid.org](mailto:mgarciasilva@salud.madrid.org) |
| Dr. Alfons Macaya Ruiz | Affiliation: University Hospital Vall d'Hebron, Barcelona 08035, Spain  Email: amacaya@vhebron.net |
| Prof. Barbara Garavaglia | Affiliation: IRCCS Foundation, Neurological Institute “Carlo Besta”, Molecular Neurogenetics, 20126 Milan, Italy  Email: segr.neurogenetica@istituto-besta.it |
| Dr. Eugenia Borgione | Affiliation: Laboratorio di Neuropatologia Clinica, U.O.S. Malattie, Neuromuscolari Associazione OASI Maria SS. ONLUS – IRCCS, Via Conte Ruggero 73, 94018 Troina, Italy  Email: eborgione@oasi.en.it |
| Dr. Simona Portaro | Affiliation: IRCCS Centro Neurolesi "Bonino Pulejo", SS113, c.da Casazza, 98124 Messina, Italy  Email: simonaportaro@hotmail.it |
| Dr. Benigno Monteagudo Sanchez | Affiliation: Hospital Arquitecto Marcide, Avenida de la Residencia S/N, Ferrol (A Coruña), 15401 Spain  Email: benims@hotmail.com |
| Dr. Richard Boles | Affiliation: Courtagen Life Sciences, 12 Gill Street Suite 3700, Woburn, MA 01801 USA  Email: Richard.Boles@courtagen.com |
| Prof. Savvas Papacostas | Affiliation: Neurology Clinic B, The Cyprus Institute of Neurology and Genetics, 6 International Airport Road, 1683 Nicosia, Cyprus  Email: savvas@cing.ac.cy |
| Dr. Michail Vikelis | Affiliation: Iatreio Kefalalgias Glyfadas, 8 Lazaraki str, 3rd floor, 16675, Athens, Greece  Email: [mvikelis@headaches.gr](mailto:mvikelis@headaches.gr) |
| Prof James Rothman | Affiliation: Department of Cell Biology, Yale School of Medicine, New Haven, CT  Email: jrothman77@yahoo.com |
| Dr Paola Giunti | Affiliation: Department of Molecular Neuroscience, University College London, London, UK  Email: [p.giunti@ucl.ac.uk](mailto:h.houlden@ucl.ac.uk) |
| Prof Henry Houlden | Affiliation: Department of Molecular Neuroscience, University College London, London, UK  Email: [h.houlden@ucl.ac.uk](mailto:h.houlden@ucl.ac.uk) |
| Dr Viorica Chelban | Affiliation: Department of Molecular Neuroscience, University College London, London, UK  Email: v.chelban@ucl.ac.uk |
| Dr. Vincenzo Salpietro | Affiliation: Department of Molecular Neuroscience, University College London, London, UK  Email: v.salpietro@ucl.ac.uk |
| Dr Emer Oconnor | Affiliation: Department of Molecular Neuroscience, University College London, London, UK  Email: e.oconnor@ucl.ac.uk |
| Stephanie Efthymiou | Affiliation: Department of Molecular Neuroscience, University College London, London, UK  Email: s.efthymiou@ucl.ac.uk |
| Prof Dimitri Kullmann | Affiliation: University College London, London, UK  Email: [d.kullmann@ucl.ac.uk](mailto:d.kullmann@ucl.ac.uk) |
| Dr Rauan Kaiyrzhanov | Affiliation: Department of Molecular Neuroscience, University College London, London, UK  Email: rauan.kaiyrzhanov.14@ucl.ac.uk |
| Roisin Sullivan | Affiliation: Department of Molecular Neuroscience, Queen's Square Institute of Neurology, UCL, London, UK.  [r.sullivan@ucl.ac.uk](mailto:r.sullivan@ucl.ac.uk) |
| Alaa Matooq Khan | Affiliation: Department of Molecular Neuroscience, Queen's Square Institute of Neurology, UCL, London, UK.  [skgtamq@ucl.ac.uk](mailto:skgtamq@ucl.ac.uk) |
| Dr Wai Yan Yau | Affiliation: Department of Molecular Neuroscience, Queen's Square Institute of Neurology, UCL, London, UK.  Email: [wai.yau.17@ucl.ac.uk](mailto:wai.yau.17@ucl.ac.uk) |
| Dr. Isabel Hostettler | Affiliation: Department of Molecular Neuroscience, Queen's Square Institute of Neurology, UCL, London, UK.  Email: [isabel.hostettler@gmail.com](mailto:isabel.hostettler@gmail.com) |
| Prof Eleni Zamba Papanicolaou | Affiliation: The Cyprus Institute of Neurology & Genetics, Nicosia, Cyprus  Email: [ezamba@cing.ac.cy](mailto:ezamba@cing.ac.cy) |
| Dr Efthymios Dardiotis | Affiliation: UNIVERSITY HOSPITAL OF LARISSA, NEUROLOGY Department, Greece  Email: [edar@med.uth.gr](mailto:edar@med.uth.gr) |
| Prof Shazia Maqbool | Affiliation: Department of Developmental and Behavioral Pediatrics, CH&ICH, Lahore, Pakistan  Email: [drshazimaq@yahoo.com](mailto:drshazimaq@yahoo.com) |
| Prof Shahnaz Ibrahim | Affiliation: Department of Pediatrics and child health, Aga Khan University, Karachi, Pakistan  Email: [shahnaz.ibrahim@aku.edu](mailto:shahnaz.ibrahim@aku.edu) |
| Prof Salman Kirmani | Affiliation: Department of Paediatrics & Child Health, The Aga Khan University, Karachi , Pakistan  Email: [salman.kirmani@aku.edu](mailto:salman.kirmani@aku.edu) |
| Dr. Nuzhat Noureen Rana | Affiliation: Department of Paediatric Neurology, Children Hospital Complex and ICH, Multan, Pakistan  Email: [drnuzhatrana@gmail.com](mailto:drnuzhatrana@gmail.com) |
| Dr. Osama Atawneh | Affiliation: Hilal Pediatric Hospital Hebron, Hebron West Bank, Palestine  Email: [osamaat@gmail.com](mailto:osamaat@gmail.com) |
| Prof Shen-Yang Lim | Affiliation: Department of Biomedical Science, Faculty of Medicine, University of Malaya, Malaysia  Email: [limshenyang@gmail.com](mailto:limshenyang@gmail.com) |
| Dr Farooq Shaikh | Affiliation: Jeffrey Cheah School of Medicine and Health Sciences, Monash University Malaysia  Email: [farooq.shaikh@monash.edu](mailto:farooq.shaikh@monash.edu) |
| Prof George Koutsis | Affiliation: Neurogenetics Unit, Neurology Department, Eginition Hospital, National and Kapodistrian University, Athens, Greece  Email: [marianthibr@med.uoa.gr](mailto:marianthibr@med.uoa.gr) |
| Dr Marianthi Breza |  |
| Prof Salvatore Mangano | Affiliation: Unità di Neuropsichiatria Infantile, AOUP "P.Giaccone" Palermo, Italy  Email: [salvatore.mangano@unipa.it](mailto:salvatore.mangano@unipa.it) |
| Dr Carmela Scuderi | Affiliation: Associazione Oasi Maria SS, 94018 Troina, Italy  Email: [cscuderi@oasi.en.it](mailto:cscuderi@oasi.en.it) |
| Dr Eugenia Borgione | Affiliation: Associazione Oasi Maria SS, 94018 Troina, Italy  Email: [eborgione@oasi.en.it](mailto:eborgione@oasi.en.it) |
| Dr Giovanna Morello | Affiliation: Institute of Neurological Sciences, National Research Council, Mangone, Italy  Email: [g.morello@isn.cnr.it](mailto:g.morello@isn.cnr.it) |
| Dr Tanya Stojkovic | Affiliation: Institute of Myology, Hôpital La Pitié Salpêtrière, Paris, France  Email: [stojkovic.tanya@aphp.fr](mailto:stojkovic.tanya@aphp.fr) |
| Dr Erin Torti | Affiliation: GenedX, Gaithersburg, Maryland, USA  Email: [etorti@genedx.com](mailto:etorti@genedx.com) |
| Prof Massimi Zollo | Affiliation: CEINGE, Biotecnologie Avanzate S.c.a.rl., Naples, Italy  Email: [massimo.zollo@unina.it](mailto:massimo.zollo@unina.it) |
| Dr Gali Heimer | Affiliation: University Hospital of Tel Aviv, Tel Aviv, Israel  Email: [galih.md@gmail.com](mailto:galih.md@gmail.com) |
| Prof Yves A. Dauvilliers | Affiliation: University Hospital Montpellier, Montpellier, France  Email: [ydauvilliers@yahoo.fr](mailto:ydauvilliers@yahoo.fr) |
| Prof Pasquale Striano | Affiliation: Institute “Giannina Gaslini”, Genova, Italy  Email: [strianop@gmail.com](mailto:strianop@gmail.com) |
| Dr Issam Al-Khawaja | Affiliation: Albashir University Hospital, Amman, Jordan  Email: [isamkhawaja61@gmail.com](mailto:isamkhawaja61@gmail.com) |
| Dr Fuad Al-Mutairi | Affiliation: King Saud University, Riyadh, Saudi Arabia  Email: [almutairifu@NGHA.MED.SA](mailto:almutairifu@NGHA.MED.SA) |
| Prof Fowzan S Alkuraya | Affiliation: King Faisal Specialist Hospital and Research Center, Riyadh, Saudi Arabia  Email: [falkuraya@kfshrc.edu.sa](mailto:falkuraya@kfshrc.edu.sa) |
| Prof Hamed Sherifa | Affiliation: Assiut University Hospital, Assiut, Egypt  Email: [hamed_sherifa@yahoo.com](mailto:hamed_sherifa@yahoo.com) |
| Dr Mie Rizig | Affiliation: Department of neuromuscular diseases, Queen square-Institute of neurology, University College London  Email: [mie.rizig@uclmail.net](mailto:mie.rizig@uclmail.net) |
| Prof. Njideka U. Okubadejo | Affiliation: College of Medicine, University of Lagos (CMUL) & Lagos University Teaching Hospital, Idi Araba, Lagos State, Nigeria |
| Dr. Oluwadamilola O. Ojo | Affiliation: College of Medicine, University of Lagos  (CMUL) & Lagos University Teaching Hospital (LUTH), Idi Araba, Lagos State, Nigeria |
| Dr. Olajumoke O. Oshinaike | Affiliation: Lagos State University College of Medicine (LASUCOM) & Lagos State University Teaching Hospital (LASUTH), Ikeja, Lagos State, Nigeria |
| Prof. Kolawole Wahab | Affiliation: University of Ilorin Teaching Hospital (UITH), Ilorin, Kwara State, Nigeria |
| Dr. Abiodun H. Bello | Affiliation: University of Ilorin Teaching Hospital (UITH), Ilorin, Kwara State, Nigeria |
| Prof. Sanni Abubakar | Affiliation: Ahmadu Bello University, Zaria, Kaduna State, Nigeria |
| Dr. Yahaya Obiabo | Affiliation: Delta State University Teaching Hospital, Oghara, Delta State, Nigeria |
| Dr. Ernest Nwazor | Affiliation: Federal Medical Centre, Owerri, Imo State, Nigeria |
| Dr. Oluchi Ekenze | Affiliation: University of Nigeria Teaching Hospital, Ituku-Ozalla, Enugu State, Nigeria |
| Dr. Uduak Williams | Affiliation: University of Calabar Teaching Hospital, Calabar, Cross Rivers State, Nigeria |
| Dr. Alagoma Iyagba | Affiliation: University of Port Harcourt Teaching Hospital, Port Harcourt, Rivers State, Nigeria |
| Dr. Lolade Taiwo | Affiliation: Babcock University, Ilishan, Remo & Federal Medical Centre, Abeokuta, Ogun State, Nigeria |
| Prof. Morenikeji Komolafe | Affiliation: Obafemi Awolowo University Teaching Hospital (OAUTH), Ile-Ife, Osun State, Nigeria |
| Dr. Olapeju Oguntunde | Affiliation: Lagos University Teaching Hospital (LUTH), Nigeria |
| Dr Sofya Pchelina | Affiliation: National Research Centre "Kurchatov Institute" B.P.Konstantinov Petersburg Nuclear Physics Institute, Russiaand Pavlov First Saint Petersburg State Medical University, Saint-Petersburg, Russia  Email: [senkkon@gmail.com](mailto:senkkon@gmail.com) |
| Dr. Konstantin Senkevich |  |
| Dr. Nourelhoda Haridy | Affiliation: Department of Neurology and Psychiatry, Assuit University Hospital, Assiut, Egypt  nourelhodaharidy@gmail.com |
| Dr. Chingiz Shashkin | Affiliation: Kazakh National State University, Almaty, Kazakhstan  Email: [chingizshashkin@gmail.com](mailto:chingizshashkin@gmail.com) |
| Dr. Nazira Zharkynbekova, | Affiliation: Shymkent Medical Academy, Kazakhstan  Email: [nazirazhar@mail.ru](mailto:nazirazhar@mail.ru) |
| Dr. Kairgali Koneyev, | Affiliation: Kazakh National State University, Almaty, Kazakhstan  Email: [kairgali@mail.ru](mailto:kairgali@mail.ru) |
| Dr. Ganieva Manizha, | Affiliation: Avicenna Tajik State Medical University, Dushanbe, Tajikistan  Email: [ganieva.manizha.79@mail.ru](mailto:ganieva.manizha.79@mail.ru) |
| Dr. Maksud Isrofilov | Affiliation: Avicenna Tajik State Medical University, Dushanbe, Tajikistan  Email:dr.maks-55@mail.ru |
| Dr. Ulviyya Guliyeva, | Affiliation: Mediclub clinic, Baku, Azerbaijan  Email: [doctor.ulya@gmail.com](mailto:doctor.ulya@gmail.com) |
| Dr. Kamran Salayev | Affiliation: Azerbaijan State Medical University, Baku, Azerbaijan  Email: [ksalayev@yahoo.com](mailto:ksalayev@yahoo.com) |
| Dr. Samson Khachatryan | Affiliation: "Somnus" Neurology Clinic Sleep and Movement Disorders Center, Yerevan, Armenia.  Email: [drsamkhach@gmail.com](mailto:drsamkhach@gmail.com) |
| Dr. Salvatore Rossi | Affiliation: Institute of Neurology, Università Cattolica del Sacro Cuore, Rome, Italy  Email: [salvatorerossi309@gmail.com](mailto:salvatorerossi309@gmail.com) |
| Prof. Gabriella Silvestri | Affiliation: Institute of Neurology, Università Cattolica del Sacro Cuore, Rome, Italy  Email: [gabriella.silvestri@unicatt.it](mailto:gabriella.silvestri@unicatt.it) |
| Dr. Thomas Bourinaris | Affiliation: Department of Molecular Neuroscience, University College London, London, UK  Email: [thomas.bourinaris.17@ucl.ac.uk](mailto:thomas.bourinaris.17@ucl.ac.uk) |
| Dr. Georgia Xiromerisiou | Affiliation: Department of Neurology, Medical School, University of Thessaly, Larissa, Greece  Email: [geoksirom@med.uth.gr](mailto:geoksirom@med.uth.gr), [georgiaxiromerisiou@gmail.com](mailto:georgiaxiromerisiou@gmail.com) |
| Dr. Liana Fidani | Affiliation: Department of Biology, Medical School, Aristotle University, Thessaloniki, Greece  Email: [lfidani@med.auth.gr](mailto:lfidani@med.auth.gr) |
| Dr. Cleanthe Spanaki | Affiliation: Department of Neurology, Medical School, University of Crete, Heraklion, Greece  Email: [kliospanaki@med.uoc.gr](mailto:kliospanaki@med.uoc.gr) |
| Dr Arianna Tucci | Affiliation: William Harvey Research Institute, The NIHR Biomedical Research Centre at Barts, Queen Mary University London, London, UK  Email: a.tucci@qmul.ac.uk |
